# Gene Expression and DNA Methylation Profiling Suggest Potential Biomarkers for Azacitidine Resistance in Myelodysplastic Syndrome

**DOI:** 10.3390/ijms25094723

**Published:** 2024-04-26

**Authors:** Da Yeon Kim, Dong-Yeop Shin, Somi Oh, Inho Kim, Eun Ju Kim

**Affiliations:** 1 Division of Radiation Biomedical Research, Korea Institute of Radiological and Medical Sciences, Seoul 01812, Republic of Korea; kimd1106@daum.net; 2 Department of Radiological and Medico-Oncological Sciences, University of Science and Technology, Daejeon 34113, Republic of Korea; 3 Cancer Research Institute, Seoul National University College of Medicine, Seoul 03080, Republic of Korea; shindongyeop@snuh.org (D.-Y.S.); sororom@hanmail.net (S.O.); 4 Center for Medical Innovation, Biomedical Research Institute, Seoul National University Hospital, Seoul 03080, Republic of Korea; 5 Division of Hematology and Medical Oncology, Department of Internal Medicine, Seoul National University Hospital, Seoul 03080, Republic of Korea; 6 Institute for Molecular Bioscience, The University of Queensland, Carmody Rd., St Lucia, Brisbane, QLD 4072, Australia; 7 Genomics and Machine Learning Lab, QIMR Berghofer Medical Research Institute, Herston Rd., Herston, Brisbane, QLD 4006, Australia

**Keywords:** myelodysplastic syndrome, azacitidine, resistance, DNA methylation, biomarkers

## Abstract

Myelodysplastic syndrome/neoplasm (MDS) comprises a group of heterogeneous hematopoietic disorders that present with genetic mutations and/or cytogenetic changes and, in the advanced stage, exhibit wide-ranging gene hypermethylation. Patients with higher-risk MDS are typically treated with repeated cycles of hypomethylating agents, such as azacitidine. However, some patients fail to respond to this therapy, and fewer than 50% show hematologic improvement. In this context, we focused on the potential use of epigenetic data in clinical management to aid in diagnostic and therapeutic decision-making. First, we used the F-36P MDS cell line to establish an azacitidine-resistant F-36P cell line. We performed expression profiling of azacitidine-resistant and parental F-36P cells and used biological and bioinformatics approaches to analyze candidate azacitidine-resistance-related genes and pathways. Eighty candidate genes were identified and found to encode proteins previously linked to cancer, chronic myeloid leukemia, and transcriptional misregulation in cancer. Interestingly, 24 of the candidate genes had promoter methylation patterns that were inversely correlated with azacitidine resistance, suggesting that DNA methylation status may contribute to azacitidine resistance. In particular, the DNA methylation status and/or mRNA expression levels of the four genes (AMER1, HSPA2, NCX1, and TNFRSF10C) may contribute to the clinical effects of azacitidine in MDS. Our study provides information on azacitidine resistance diagnostic genes in MDS patients, which can be of great help in monitoring the effectiveness of treatment in progressing azacitidine treatment for newly diagnosed MDS patients.

## 1. Introduction

Myelodysplastic syndrome/neoplasm (MDS) is a clonal hematopoietic stem cell (HSC) disease that mainly involves cytogenetic changes and/or genetic mutations and, in advanced stages, exhibits widespread gene hypermethylation [1]. The main features of MDS are myeloid cell cytopenias, morphologic dysplasia, ineffective hematopoiesis, and a high risk of transformation into acute myeloid leukemia (AML) [2]. MDS mainly affects elderly patients. Although the actual epidemiology of MDS is unknown, its incidence is on the rise due to the growing population of aged individuals, the increasing use of cytotoxic agents in treating diseases, and exposure to environmental carcinogens such as organic solvents [3].

Currently, only two treatment modalities can improve overall survival in high-risk MDS: allogeneic stem cell transplantation and treatment with the hypomethylating agents (HMAs) azacitidine (AZA) or decitabine. Allogeneic stem cell transplantation is the only potentially curative therapy for high-risk MDS. However, due to advanced patient age, medical comorbidities, and the limited availability of stem cell donors, relatively few patients actually undergo stem cell transplants. For patients with higher-risk MDS who are not fit for intensive approaches, HMA treatment has become the standard of care [4,5]. AZA was first approved by the Food and Drug Administration (FDA) for treating MDS in 2004. It received expanded approval in 2008 for patients with higher-risk MDS based on the Phase III AZA-001 trial; this large, randomized trial showed a greater median overall survival (OS) of 24.5 months for AZA-treated patients compared to that of 15.0 months for patients receiving supportive care [6].

AZA has been shown to prolong patient survival, improve clinical outcomes and quality of life, and delay progression to AML [7]. However, only 30% to 40% of patients will respond to therapy; of them, most will achieve hematologic improvement (HI) in blood counts, but only 10% to 15% will achieve a complete response (CR), which is the response criterion most reliably associated with improvement in OS [8]. There are few alternative treatment options for patients who fail to respond to AZA, and their prognosis is extremely poor [9]. To date, the mechanisms underlying HMA resistance are poorly understood [10], and we lack a reliable method for predicting the likelihood that a patient will benefit from HMA treatment [11]. Possible predictive factors for HMA responsiveness have been extensively investigated in recent studies, but the conclusions are controversial, and a consensus has not been reached [12,13,14]. Clinical variables and patient characteristics have not consistently predicted HMA responsiveness, and although recurrent somatic mutations have been described for several genes in MDS and appear to have implications for disease biology and OS, their impacts on HMA responsiveness remain debated [8]. Therefore, there is an unmet need for biomarkers that can accurately predict HMA responsiveness or resistance [15].

MDS occurs in cases of abnormal gene expression due to genetic mutations or epigenetic events. Given the role of DNA methylation in MDS pathogenesis, it is a potential candidate [15,16]. It has been suggested that increased expression of maintenance DNA methyltransferases (DNMTs) or de novo expression of specific DNMTs (e.g., DNMT3A and DNMT3B) contribute to the development of leukemia by inducing aberrant hypermethylation of important genomic regions [17]. MDS is typically characterized by global hypermethylation, potentially explaining why MDS patients respond well to HMAs. Overall, global DNA methylation levels and site-specific methylations have shown promise as potential biomarkers for predicting HMA resistance in MDS [15].

The aim of this study was to identify genetic and/or epigenetic profiles that can be successfully translated for use as biomarkers to predict the AZA resistance of MDS patients. We focused on the association between differential gene promoter methylation and AZA resistance. We hope that the identified four biomarker candidates (AMER1, HSPA2, NCX1, and TNFRSF10C) will clinically facilitate the early identification of AZA resistance, thus avoiding months of potentially futile therapy, and ultimately promote the development of novel strategies for prognostic prediction and personalized therapy for MDS patients.

## 2. Results

### 2.1. Levels of DNMTs and Methyl-Binding Proteins Are Higher in F-36P/AZA Cells than in F-36P Cells

**Figure 1:**
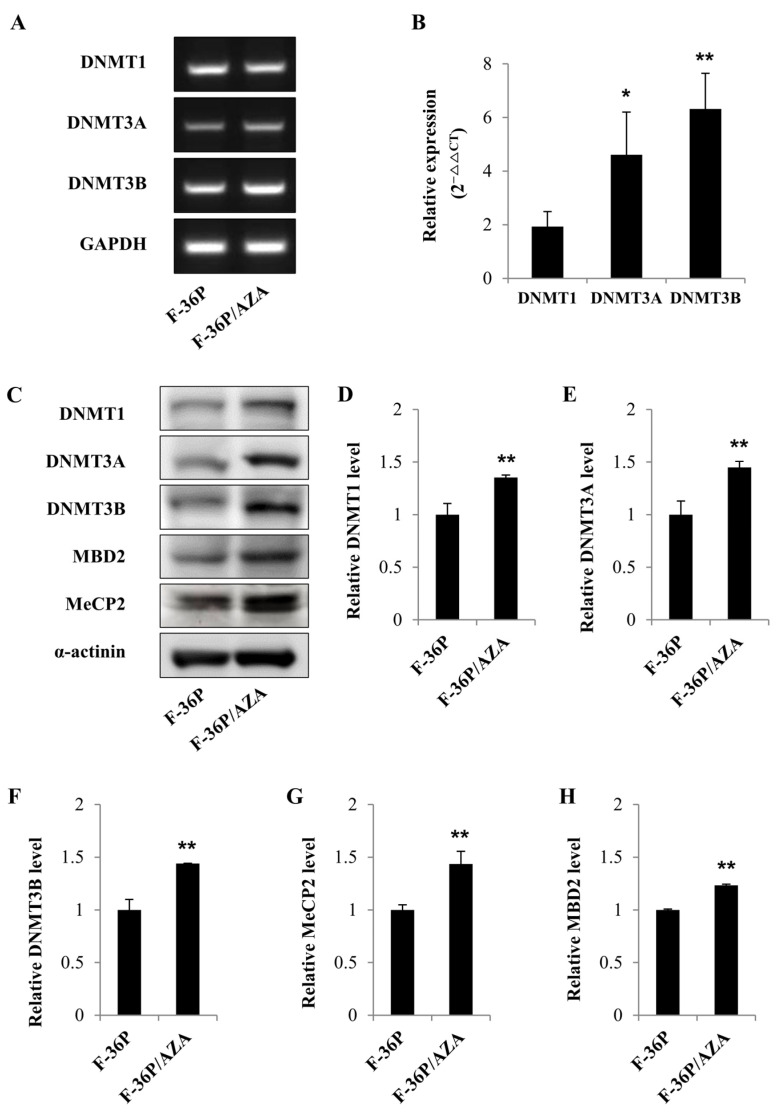
Gene expression and protein levels of DNMTs in F-36P and F-36P/AZA cells. (**A**) The mRNA expression levels of DNMTs in F-36P and F-36P/AZA were detected via PCR. (**B**) RT-qPCR was applied to assess the mRNA expression levels of DNMTs in F-36P and F-36P/AZA cells, with GAPDH used as the loading or internal control. The data were analyzed using the 2^−ΔΔCT^ method. (**C**) The protein levels of DNMTs, MBD2, and MeCP2 were determined via Western blotting, with α-actin used as the loading control. (**D**–**H**) The protein levels of DNMTs, MBD2, and MeCP2 in cell lysates were semi-quantified relative to the control group. Data are presented as mean ± SD (*n* = 3); * *p* < 0.05 and ** *p* < 0.01 vs. Control.

To study the mechanisms underlying AZA resistance, we established an in vitro AZA-resistant cell model from the parental cell line F-36P. The F-36P/AZA cell model generated for these studies was significantly resistant to AZA and has characteristics that differ from those of F-36P cells (Figure S1). The IC50 values for AZA were 1 μmol/L in F-36P cells and 125 μmol/L in F-36P/AZA cells (constituting a 125-fold increase compared to the parental cell line) (Figure S1D). Previous mechanistic studies of AZA resistance in human leukemia cell lines found that the mRNA levels of the genes encoding DNMT1, DNMT3a, and DNMT3b were increased in HMA-resistant cells [18]. To confirm this and provide further insight into the mechanism underlying AZA resistance, we compared DNMT levels in F-36P and F-36P/AZA cells. PCR and qRT-PCR revealed that the mRNA expression levels of DNMTs were higher in F-36P/AZA cells compared with those in F-36P cells (Figure 1A,B). Among the DNMTs, the expression level of DNMT3B was higher than the expression levels of DNMT1 and DNMT3A. Western blot analysis of DNMTs, MBD2, and MeCP2 revealed that the tested proteins were expressed at higher levels in F-36P/AZA cells than in F-36P cells (Figure 1C–H). These analyses showed that, as expected, the levels of DNMTs were significantly upregulated in F-36P/AZA cells compared with F-36P cells.

### 2.2. Identification of Genes Exhibiting Differential Expression between F-36P/AZA and F-36P Cells

**Figure 2:**
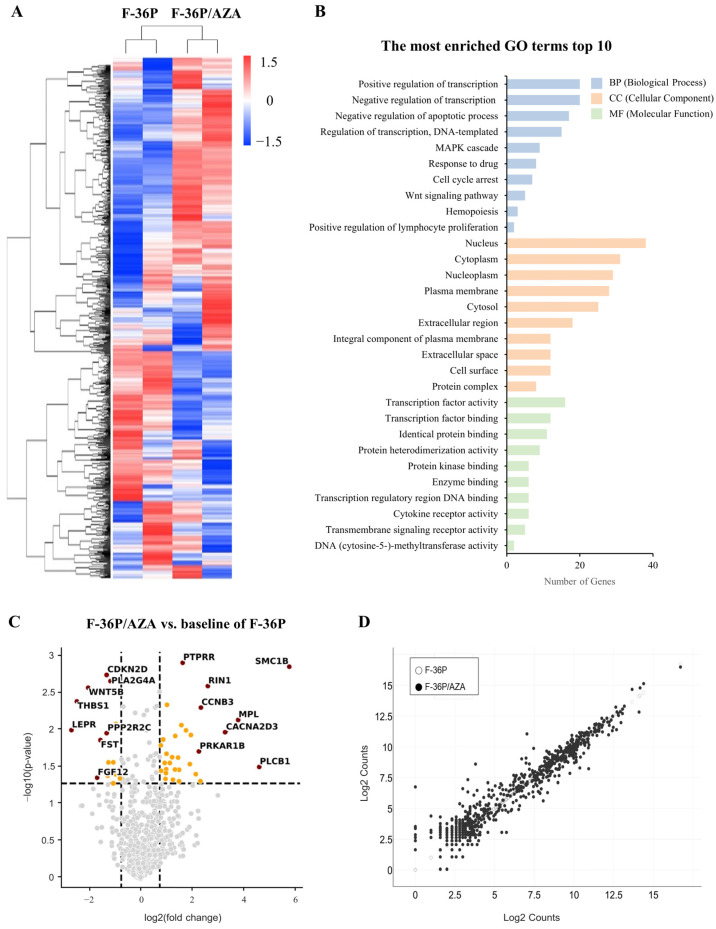
Analysis of differentially expressed genes (DEGs) in F-36P/AZA cells compared to F-36P cells. (**A**) Heatmap illustrates 770 genes showing significantly different expression levels (in column) in a NanoString (RNA-seq) analysis of F-36P versus F-36P/AZA cells. The colors in the boxes indicate the gene expression level. Representations of genes were processed using the general linear model likelihood ratio test (*p* < 0.05 and absolute log2 fold change > 1). (**B**) Top 10 Gene Ontology (GO) terms identified in the GO analysis. The vertical portion presents the GO terms, while the horizontal and the length of the graph represent the gene numbers. The colors in the graph denote the different GO categories. (**C**) DEGs between F-36P and F-36P/AZA cells are shown as log10 (*p*-value) versus log2 (fold change) and presented graphically as volcano plots. DEGs are indicated in yellow circle, and Top DEGs are indicated in red circle. The horizontal lines indicate statistical significance at *p* < 0.05. (**D**) Scatter plot of DEGs in F-36P/AZA versus F-36P cells. DEGs were selected according to the following criteria: (LogFC) ≥ 1.5 and *p* < 0.05.

To identify candidate AZA resistance genes, we searched for candidate genes that were differentially expressed in F-36P versus F-36P/AZA cells. NanoString analysis [19] was used to identify differentially expressed genes (DEGs), which were visualized using nSolver as a heatmap, a volcano plot, and a scatter plot. To generate the cluster heatmap [20], we used hierarchical clustering with average linkage and a Euclidean distance metric (Figure 2A). A gene ontology (GO) analysis [21] of upregulated DEGs was used to conceptualize the possible involved functions of the encoded products (Figure 2B and Table S3). To display the DEG distribution in F-36P/AZA cells versus F-36P cells, we used a volcano plot (Figure 2C and Table S4) and a scatter plot (Figure 2D). Collectively, the obtained data allowed us to focus our basic database investigations.

**Figure 3:**
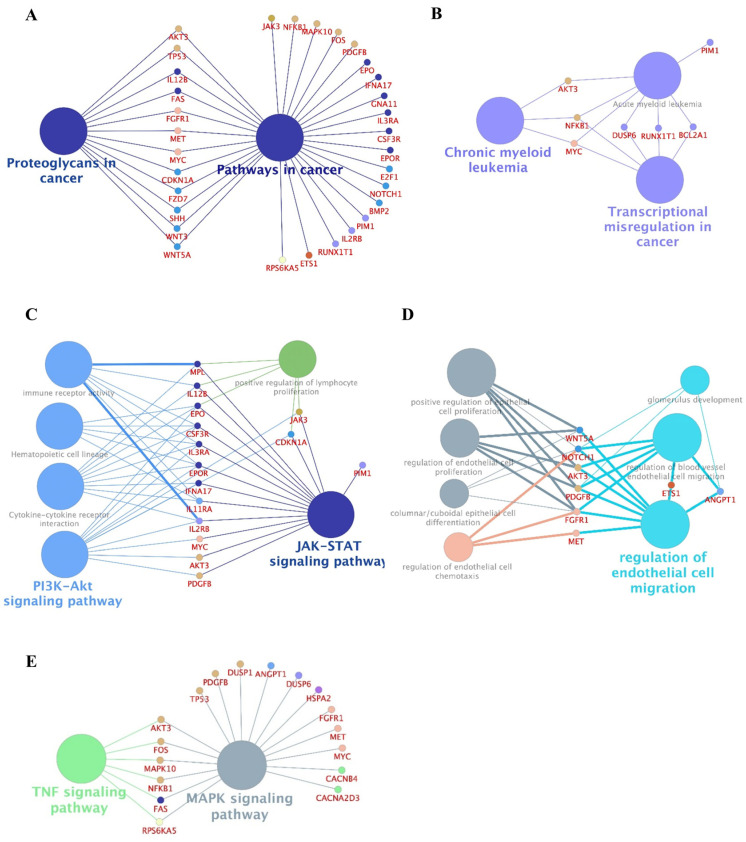
ClueGO-analysis-based enrichment maps derived from GO terms associated with DEGs. (**A**–**E**) Highly interconnected GO terms are presented here. These images show the most significant functional categories identified by ClueGO Cytoscape plugin and illustrate how terms are grouped and interact with each other. Bold font indicates the top GO terms. Gene names within subgroups were generated using ClueGO default settings. The node size indicates the significance of enrichment. The node colors correspond to degrees of similarity, with nodes of the same color belonging to the same cluster (pathway). All GO terms shown are statistically significant (*p* < 0.05 with Bonferroni correction).

Protein–protein interaction (PPI) networks can be used to identify physical contact between protein pairs and highlight small biological pathway subsets [22]. Here, we used 80 DEGs to generate a PPI network containing 80 nodes and 948 edges (Figure S2A and Table S5). From this PPI network, we screened the hub networks with MCODE [23] and CytoHubba [24] (Figure S2B–G) and applied ClueGO to analyze and visualize the interrelationships of the enriched pathways and DEGs [25]. This analysis suggested various AZA resistance-linked processes, including pathways involved in cancer, chronic myeloid leukemia, transcriptional misregulation in cancer, regulation of endothelial cell migration, JAK-STAT signaling, and MAPK signaling (Figure 3).

### 2.3. Evaluation of Candidate Genes via qRT-PCR and Pyrosequencing

**Figure 4:**
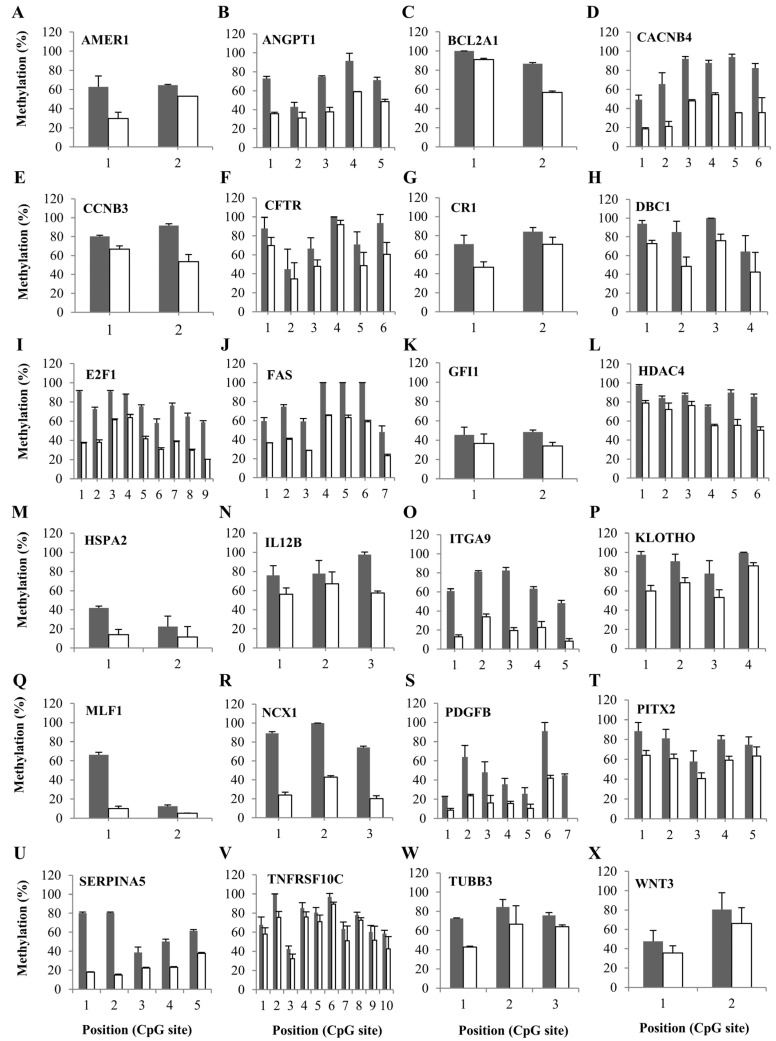
DNA methylation status of candidate genes. (**A**–**X**) Methylation levels of the selected candidate genes in F-36P and F-36P/AZA cells were detected using bisulfite sequencing. The DNA methylation status is expressed as a percentage of CpG methylation. F-36P-expressed genes are marked in gray, and F-36P/AZA-expressed genes are given in white. Differential expression was considered significant at *p* < 0.05. Error bars indicate standard deviations (*n* = 3).

We next used quantitative RT-PCR analysis to verify the top 80 DEGs with the most fold-changes (Figure S3). A subset of the selected DEGs encoded proteins involved in pathways related to cancer (e.g., FAS and PIM1), chronic myeloid leukemia (e.g., AKT3 andMYC), transcriptional misregulation in cancer (e.g., RUNXT1 and BCL2A1), the regulation of endothelial cell migration (e.g., PDGFB and NOTCH1), JAK-STAT signaling (e.g., MPL and JAK3), and MAPK signaling (e.g., HSPA2 and CACNB4). The molecular properties of these gene products are summarized in the Supplementary Data (Table S6). We examined the DNA methylation levels of 80 genes in F-36P and F-36P/AZA cells and found that F-36P/AZA cells consistently exhibited lower methylation than F-36P cells in 24 genes (Figure 4). This may explain the enhanced expression of these 24 candidate genes in F-36P/AZA cells and suggests that DNA methylation changes in these candidate genes could be used to predict AZA responsiveness in MDS.

### 2.4. Comparison of Gene Expression in Bone Marrow from Patients with MDS

Bone marrow-derived blood samples from four patients with MDS at the time of the first response evaluation after four cycles of azacitidine treatment were included in this study. The median age of patients at the time of MDS diagnosis was 61 years (range, 47–78). The MDS subtypes and corresponding affected patient numbers were as follows: two patients with MDS-SLD and one each with MDS-EB1 and MDS-EB2. The risk categories at baseline were low/intermediate-1 according to IPSS and low/high according to IPSS-R (Table 1). The patients were treated with a median of 37.5 cycles of AZA (range, 4–46). Three patients (patients 2–4) achieved objective responses (complete remission), while one patient (patient 1) did not show any objective response after four cycles of AZA treatment (stable disease). Patient 1 expired due to anemia despite undergoing allogeneic hematopoietic stem cell transplantation. The duration of response was 6 months for patient 2, 46 months for patient 3, and 74 months for patient 4. The survival periods were 84 months for patient 1, 12 months for patient 2, and 45 months for patient 3. Patient 4 has continued to respond to AZA for more than 6 years and is alive at this time. The IPSS-R was correlated with time to (leukemia) progression and overall survival, as previously reported [26].

**Figure 5:**
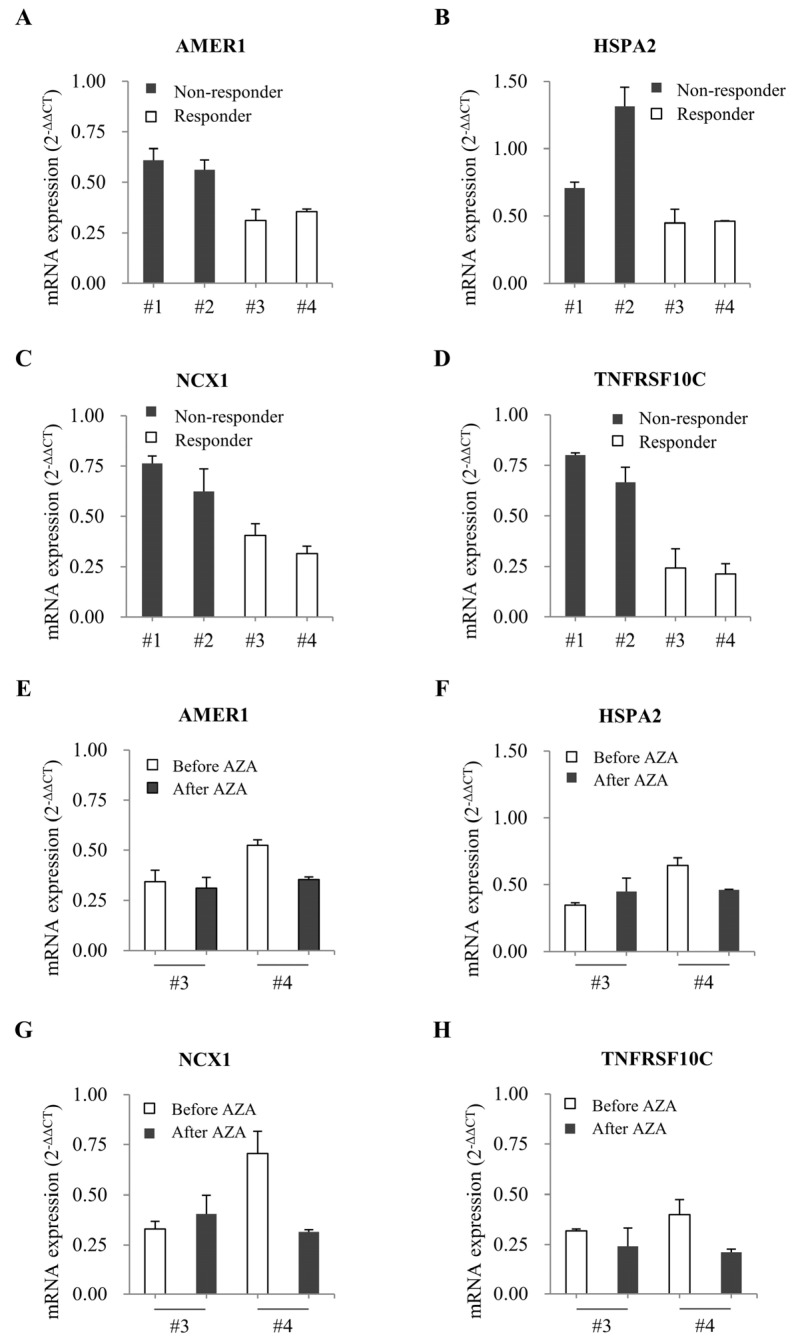
Comparison of gene expression in MDS patients before and after AZA treatment. (**A**–**D**) The graphs show the mRNA levels of candidate genes in the bone marrow of MDS patients after AZA treatment. (**E**–**H**) The graphs show the mRNA levels of candidate genes in the bone marrow of MDS patients before (white) and after (black) AZA treatment. GAPDH was used as the loading or internal control, and the results were analyzed using the 2^−ΔΔCT^ method. Differential expression was considered significant at *p* < 0.05. Error bars indicate standard deviations (*n* = 3).

We next reviewed the cBioPortal [27] and UALCAN databases for relevant information on the identified candidate genes. Our cBioPortal review revealed that the genes were associated with different types of alterations (mutation, amplification, and deletion) (Figure S4), while our UALCAN results showed that their higher-level expression was associated with poor prognosis in AML (Figure S5). qRT-PCR analysis of candidate gene expression levels in bone-marrow-derived samples obtained from patients 1–4 after four cycles of AZA treatment revealed significant expression differences for four of the twenty-four genes: as shown in Figure 5A–D, the levels of mRNAs encoding AMER1, HSPA2, NCX1, and TNFRSF10C were higher in patients 1 (with no response to AZA) and 2 (with a 6-month short-duration response to AZA) compared to those in patients 3 (with a prolonged objective response of more than 3 years) and 4 (with a response of more than 6 years). These results suggest that the expression levels of these four candidate genes may differ significantly between patients with short- and long-duration responses to AZA. Bone-marrow-derived blood samples before the azacitidine treatment were available for two of the four patients (patients 3 and 4). So, we also performed qRT-PCR on pre-AZA-treatment samples from patients 3 and 4 (Figure 5E–H) and found that, interestingly, there was no difference in the expression levels of the four abovementioned genes in the samples obtained from patients 3 and 4 before and after AZA treatment.

### 2.5. DNA Methylation Status in Bone Marrow from Patients with MDS

**Figure 6:**
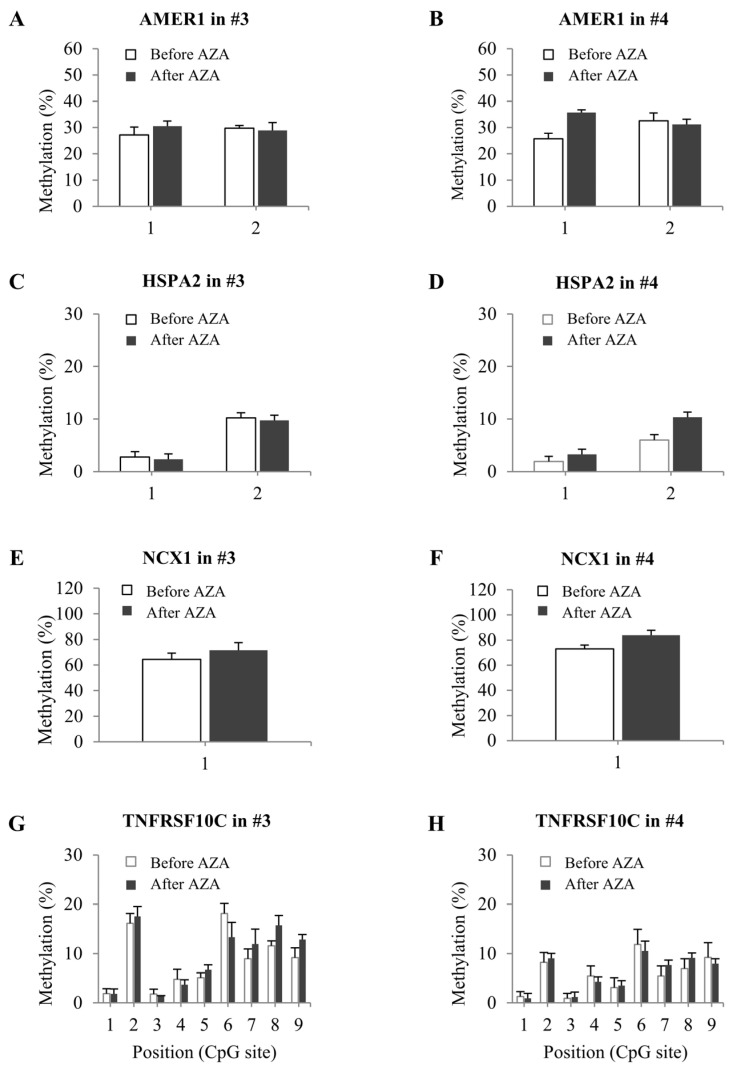
DNA methylation status of MDS patients before and after AZA treatment. (**A**–**H**) Methylation levels of four selected candidate genes in patients 3 and 4 before (white) and after (black) AZA treatment were detected via bisulfite sequencing and expressed as a percentage of CpG methylation. Differential expression was considered significant at *p* < 0.05. Error bars indicate standard deviations (*n* = 3).

We next performed pyrosequencing to assess the DNA methylation statuses in samples obtained from patients 3 and 4 before and after AZA treatment. We did not observe any significant change in the DNA methylation of these four genes following AZA treatment in these patients (Figure 6). This may explain why there was no difference in the mRNA expression of these genes in the same samples.

## 3. Discussion

Epigenetic disorders are an important factor in the pathophysiology of MDS, and abnormal DNA methylation has been repeatedly identified as a key event in this disease [28,29]. Promoter hypermethylation of tumor suppressor genes is involved in promoting the survival, growth, and metastasis of cancer, while abnormal hypomethylation can lead to the transcriptional activation of tumor genes in various cancers, including MDS [29,30]. Based on the hypothesis that hypermethylation might favor leukemogenesis by silencing tumor suppressor genes, demethylating agents, including AZA, have been studied for their ability to antagonize this process and thereby treat MDS [31]. AZA has been used effectively to treat MDS and AML for more than a decade [32], but the AZA response rate is about 50%, and the median response period is less than 18 months [33]. Because the underlying resistance mechanisms are not well understood, it is difficult to accurately predict which patients will respond [34]. Thus, it is important to improve our understanding of the mechanisms leading to AZA failure and identify and validate biomarkers that can predict treatment responses.

Demethylation and upregulation of cancer genes after HMA treatment are associated with poor outcomes among high-risk MDS patients [29,35,36], and abnormal DNA methylation is known to contribute to tumor progression, metastasis, and resistance to currently available tumor treatment [37,38]. These findings support the notion that epigenetic changes are the driving force behind the acquisition of cancer drug resistance. Hypermethylation and hypomethylation of tumor suppressor genes and tumor gene-regulatory domains have been tested as possible biomarkers for cancer risk, diagnosis, and/or prognosis. In addition, methylation status analysis of certain genes can be useful in guiding the selection of cancer chemotherapy, immunotherapy, or targeted therapy [38]. Thus, biomarkers targeting DNA hypomethylation may be useful for predicting resistance to AZA in MDS patients [29]. However, the cancer gene upregulation and/or reactivation typical of the acquisition of AZA resistance in MDS have not been well described.

In studies seeking to identify biomarkers that can predict HMA responsiveness in hematological diseases, numerous researchers have explored somatic cell gene mutations and acquired mutations [39]. The TET2, TP53, IDH1, IDH2, and DNMT3A genes are frequently mutated in myeloid malignancies [40,41] and have been correlated with increased or decreased responsiveness [34]. However, inconsistent results have been reported with respect to the predicted and prognostic values of various mutations [39,42]. The International Prognostic Scoring System (IPSS) and the revised IPSS (IPSS-R), encompassing a combination of cytogenetic and clinical data, and the recent IPSS-M incorporating molecular mutation data [43] are used to screen patients for treatment, but these approaches are incomplete and do not predict who will respond to AZA or other treatment modalities in MDS. Joint efforts to supplement the IPSS using gene expression profiles, DNA methylation profiles, and/or high-resolution chromosome analysis should critically contribute to further progress in understanding and treating this disease [44].

To test the hypothesis that AZA resistance can induce the demethylation (or upregulation) of tumor genes, we identified AZA-resistance-associated DEGs by comparing 770 tumor genes in F-36P versus F-36P/AZA cells. We then selected 80 candidate DEGs, including those involved in pathways related to cancer, transcriptional misregulation in cancer, PI3K-Akt signaling, JAK-STAT signaling, and MAPK signaling. Interestingly, 24 genes were shown to be associated with different types of changes, and 8 of these genes (CFTR, TUB3, GFI1, CR1, CCNB3, BCL2A1, AMER1, and PITX2) were amplified in the AZA-resistant cell line. We examined the prognostic significance of the mRNA expression levels of the 24 genes using the UALCAN database and found that high-level expression of 10 of the genes (AMER1, BCL2A1, CCNB3, CR1, HSPA2, ITGA9, MLF1, NCX1, PDGFB, and WNT3) was associated with lower overall survival in AML patients. These results suggest that most of our candidate genes are clinically correlated.

Finally, we obtained bone marrow from MDS patients and tested to see if these 24 biomarker candidates could be used to predict their AZA resistance. The best response of patient 1 was NR, while those of patients 2–4 had a CR; however, we observed candidate gene expression differences even between patients exhibiting CR. In particular, patients exhibiting an upregulation of the genes encoding APC membrane recruitment protein 1 (AMER1), heat-shock-related 70-kDa protein 2 (HSPA2), solute carrier family 8 member A1 (SLC8A1), and TNF receptor superfamily member 10c (TNFRSF10C) had worse outcomes. Assessment of these four genes expression showed that, interestingly, there was no significant difference in the samples obtained from patients 3 and 4 before and after AZA treatment. There was also no difference in the DNA methylation statuses of these four genes in patients 3 and 4. By combining expression data and prognostic information from a survival curve, we observed negative correlations between the DNA methylation and RNA expressions levels of the four key genes. Moreover, their RNA expression levels among the responder and non-responder had corresponding prognoses.

This is the first study to comprehensively investigate the associations between epigenetic modalities and gene expression in AZA-resistant MDS cells. We identified genetic and epigenetic profiles that can be successfully translated into biomarkers capable of predicting the statuses of MDS patients with resistance to AZA. We conclude that the observed gene upregulation could be explained by epigenetic changes and propose that AMER1, HSPA2, NCX1, and TNFRSF10C may contribute to the clinical effects of AZA in MDS. However, as we studied a relatively small group of MDS patients, further studies involving larger numbers of patients should be performed to clarify whether the identified genes can be assessed as a means of predicting AZA resistance. In addition, our patients cannot accurately represent MDS as a whole since there were no patients with MDS-specific cytogenetic abnormalities in our cohort. The role of next-generation sequencing in MDS with a normal karyotype is already well known [45]. Unfortunately, we could not perform NGS to detect recurrent mutations in MDS for our patient cohort. However, our patients could be representative of MDS with a normal karyotype. We hope that the biomarker candidates identified here will serve as a clinically useful tool for the early identification of azacitidine resistance.

## 4. Materials and Methods

### 4.1. AZA-Resistant Cell Selection and Culture

The F-36P human leukemia (MDS) cell line [46] was obtained from European Collection of Authenticated Cell Cultures (ECACC, Salisbury, UK). The AZA-resistant F-36P cell line, F-36P/AZA, was generated by treating the parental F-36P cells with incrementally increasing concentrations of AZA (Sigma-Aldrich, St. Louis, MO, USA) ranging from 1 μM to 125 μM. Selected cells were cultured in AZA-free medium for at least 2 weeks before being used in experiments. F-36P and F-36P/AZA cells were cultured in RPMI-1640 medium (GenDEPOT, Katy, TX, USA) containing 5% fetal bovine serum (GenDEPOT), 1% penicillin (GenDEPOT), 5 ng/mL of interleukin (IL)-3 (Sigma-Aldrich), and 2 mM of glutamine (Sigma-Aldrich). Cells were maintained at 37 °C in an atmosphere containing 5% CO_2_.

### 4.2. RNA Isolation

Total RNA was isolated with QIAzol reagent (Qiagen, Hilden, Germany), following the manufacturer’s instructions. The quality of total RNA was checked via on-chip electrophoresis using an Agilent 2100 Bioanalyzer (Agilent Technologies, Santa Clara, CA, USA), and the RNA concentration was determined with a NanoDrop 2000 Spectrophotometer (ND-2000; Thermo Fisher Scientific Inc., Waltham, MA, USA).

### 4.3. Quantitative Reverse Transcription-PCR (qRT-PCR)

For mRNA detection, the extracted RNA was reverse-transcribed into cDNA using amfiRivert reverse transcriptase (GenDEPOT) according to the manufacturer’s instructions. qRT-PCR was performed via a Mic Real-Time PCR system (Bio Molecular Systems, Upper Coomera, QLD, Australia) using Luna Universal qPCR master mix (New England Biolabs Inc., Ipswich, MA, USA). GAPDH was used as the internal control for normalizing the expression of target genes. The results were calculated using the 2^−ΔΔCT^ method. The utilized primer sequences are listed in Table S1.

### 4.4. Western Blotting

Cells were lysed with lysis buffer (GenDEPOT). Equal amounts of protein were resolved using 8% sodium dodecyl sulfate-polyacrylamide gel electrophoresis (SDS-PAGE), and the resolved proteins were transferred to nitrocellulose membranes. The membranes were blocked via incubation with 5% non-fat dry milk in PBS containing 0.1% Tween-20 (PBST) for 1 h and then incubated with primary antibodies overnight at 4 °C. After three washes with PBST, the blots were incubated with horseradish peroxidase (HRP)-conjugated secondary antibodies (GenDEPOT) for 30 min at room temperature. Protein bands were detected using Western Lightning Plus ECL (Perkin Elmer, Waltham, MA, USA) and imaged with an Amersham Imager 600 (GE Healthcare, Piscataway, NJ, USA). Primary antibodies against DNMT1 (ab13537), DNMT3a (ab13888), DNMT3b (ab13604), MBD2 (ab38646), and MeCP2 (ab2828) were obtained from Abcam (Abcam, Cambridge, UK), while a primary antibody against α-actinin (sc-17829) was purchased from Santa Cruz Biotechnology (Santa Cruz, CA, USA).

### 4.5. NanoString Gene Expression Analysis

Gene expression was measured using the nCounter PanCancer Pathways Panel via a NanoString platform (NanoString Technologies, Seattle, WA, USA). The PanCancer Pathways Panel consists of 770 genes, including 20 housekeeping genes. For each hybridization reaction, 100 ng of total RNA was used. After hybridization, the samples were loaded into an nCounter Cartridge and processed via the nCounter Digital Analyzer for quantification of the target mRNA in each sample. Quality control and normalization of raw gene expression counts were performed with nSolver Analysis Software Version 4.0 (NanoString Technologies). Data mining and graphic visualization were performed using nCounter Advanced Analysis Software Version 2.0 (NanoString Technologies).

### 4.6. Gene Ontology (GO) and Pathway Enrichment Analyses of Differentially Expressed Genes (DEGs)

Genes exhibiting differential expression between F-36P and F-36P/AZA samples were identified via NanoString analysis. *p* < 0.05 and Log2 (fold change) ≥ 1 were used as the cut-off criteria for identifying significant DEGs. GO enrichment analysis of DEGs was performed using DAVID (version 6.8; Database for Annotation, Visualization and Integrated Discovery; https://david.ncifcrf.gov/ accessed on 19 December 2022). This web-based platform for gene functional annotations and biological meaning elucidation classifies DEGs by the attributes of molecular function (MF), biological process (BP), and cellular components (CCs). Pathway enrichment analysis of DEGs was generated using the Kyoto Encyclopedia of Genes and Genomes (KEGG) component of the DAVID website, with *p* < 0.05 utilized as the threshold value.

### 4.7. Protein–Protein Network and Module Analyses

To clarify the relationships among proteins encoded by selected genes, we established a protein–protein interaction (PPI) network using Cytoscape (version 3.9.1; https://cytoscape.org/ accessed on 10 January 2023). The PPI network was built based on the STRING database of Cytoscape, and PPI pairs with minimum interaction scores > 0.4 were extracted. Hub genes of the PPI network were identified according to the Maximal Clique Centrality (MCC) methods, using the cytoHubba plugin of Cytoscape. GO and KEGG enrichment analyses (functional analyses) of selected genes were performed using the ClueGO plugin of Cytoscape. A *p*-value < 0.05 was considered to be indicative of a statistically significant difference.

### 4.8. DNA Extraction and Bisulfite Conversion

Genomic DNA (gDNA) was extracted using a QIAamp DNA Mini Kit (Qiagen) according to the manufacturer’s protocol, and DNA quality was assessed using NanoDrop 2000 Spectrophotometer (ND-2000; Thermo Fisher Scientific Inc.). A total of 1 μg of DNA was subjected to bisulfite conversion by using an EZ DNA Methylation kit (Zymo Research, Orange, CA, USA) according to the manufacturer’s instructions. The converted DNA was used as a template for methylation-specific PCR.

### 4.9. Quantitative Methylation Analysis via Pyrosequencing

The converted DNA was amplified by using a PyroMark PCR kit (Qiagen) according to the manufacturer’s instructions. The utilized primers (Table S2) were designed using the PyroMark Assay Design Software Version 2.0 (Qiagen). The PCR products were resolved using 1% agarose gel electrophoresis and visualized via staining with TopRed Nucleic Acid Gel Stain (BioPure, Horndean, UK). Pyrosequencing was performed on a PyroMark Q48 Autoprep (Qiagen) using PyroMark Q48 magnetic beads (Qiagen) and the PyroMark Q48 Advanced CpG Reagent (Qiagen), as described in the manufacturer’s protocol. Methylation levels for each CpG within the targeted region were quantified using PyroMark Q48 Software Version 2.4.2 (Qiagen). Relative peak height differences were used to calculate the percentage of methylation at each site. All pyrosequencing experiments were performed three times.

### 4.10. Validation of Genetic Alterations in Candidate Genes

Genetic alterations of hub genes were additionally investigated in an extensive myeloid neoplasm dataset contained within cBioPortal (http://cbioportal.org/ accessed on 24 February 2023). This open-source platform provides visualization tools, analytic tools, and downloadable large-scale cancer genomic datasets.

### 4.11. Patient Enrollment and Treatment

Bone-marrow-derived blood samples from four patients with MDS at the time of the 1st response evaluation after the 4 cycles of azacitidine treatment were used for this study. Additionally, pre-treatment samples were also used for two of four MDS patients. The MDS patients were subtyped according to the revised World Health Organization classification of myeloid neoplasm [47]. They were treated with AZA (75 mg/m^2^/d for 7 days every 4 weeks), and response to treatment and clinical outcome were evaluated according to the revised International Working Group (IWG) response criteria. The clinical characteristics of the patients are summarized in Table 1. Bone marrow samples from nine donors with no bone marrow involvement of hematological malignancy were used as controls. Written informed consent was obtained from all tested subjects in accordance with the guidelines of the Institutional Review Board of Seoul National University Hospital (SNUH).

### 4.12. Statistical Analysis

We performed all experiments in, at least, triplicate (*n* ≥ 3). The data are presented as means ± standard deviation (SD). To analyze differences between the means of two samples, we used the Student’s *t*-test for independent samples, as applied with SPSS software version 26.0 (SPSS Inc., Chicago, IL, USA). A *p*-value < 0.05 was considered to represent a statistically significant difference.

## 5. Conclusions

In summary, we identified genetic and/or epigenetic profiles that could be used as biomarkers for predicting azacitidine resistance in MDS patients. As a result, four biomarkers (AMER1, HSPA2, NCX1, and TNFRSF10C) contributing to azacitidine resistance were discovered. These findings have the potential to serve as a clinically relevant biomarker for azacitidine resistance and a promising therapeutic target for new treatment strategies.

## Figures and Tables

**Table 1:** Clinical characteristics of the MDS patients treated with AZA.

Sample No.	1	2	3	4
Sex	Female	Male	Female	Female
Age (years)	54	78	65	47
Weight (kg)	53.2	67	66	64.5
Height (m)	1.54	1.75	1.57	1.62
BMI (Kg/m^2^)	22.4	21.9	26.8	24.6
Underlying disease	Atrial septal defect	HTN	HTN/depression	ADPKD, CKD
Baseline clinical characteristics				
WBC (×10^6^/L)	4500	3730	940	3470
ANC (×10^6^/L)	1260	1828	190	2110
Hb (g/dL)	9.5	8	9.5	7.4
Platelets (×10^9^/L)	249	178	31	104
BM blasts (%)	0.5	13.9	6.5	0.6
Cytogenetic abnormalities	none	none	none	none
IPSS	0	1.5	1	0
IPSS risk category	Low	Int-1	Int-1	Low
IPSS-R	2	5	5.5	2.5
IPSS-R risk category	Low	High	High	Low
MDS subtypes (WHO)	MDS-SLD	MDS-EB2	MDS-EB1	MDS-SLD
Treatment cycle of AZA	4	7	18	46
Best response	NR	CR	CR	HI
Progression (Leukemic transformation)	No	Yes	Yes	No
PFS (month)	7Y	6M	3Y10M	6Y2M
Allogeneic HSCT	Yes	No	No	No
Time to HSCT	6Y4M			
F/U period (month)	7Y	1Y	3Y9M	6Y2M
F/U result	Dead	Dead	Dead	continued response to AZA
Cause of death	Pneumonia	AML	AML	Alive

Abbreviation: BMI, Body Mass Index; HTN, hypertension; ADPKD, autosomal dominant polycystic kidney disease; CKD, chronic kidney disease; WBC, white blood cell count; ANC, absolute neutrophil count; Hb, hemoglobin; BM, bone marrow, IPSS, International Prognostic Scoring System; IPSS-R, Revised International Prognostic Scoring System; Int, intermediate; MDS-SLD, MDS with single lineage dysplasia; MDS-EB, MDS with excess blasts; NR, no response; CR, complete remission; HI, hematologic improvement; PFS, progression-free survival; HSCT, hematopoietic stem cell transplantation; F/U, follow-up.

## Data Availability

The original contributions presented in this study are included in the article/Supplementary Materials; further inquiries can be directed to the corresponding author/s.

## Supplementary Materials

The following supporting information can be downloaded at https://www.mdpi.com/article/10.3390/ijms25094723/s1.
