# Supplementary material for: Gene Expression and DNA Methylation Profiling Suggest Potential Biomarkers for Azacitidine Resistance in Myelodysplastic Syndrome
Source: Int J Mol Sci. 2024 Apr 26;25(9):4723. doi: 10.3390/ijms25094723 (PMC11083267; doi:10.3390/ijms25094723)
Supplement: Supplementary file 1 [file ijms-25-04723-s001.zip › ijms-2960878-supplementary.pdf]

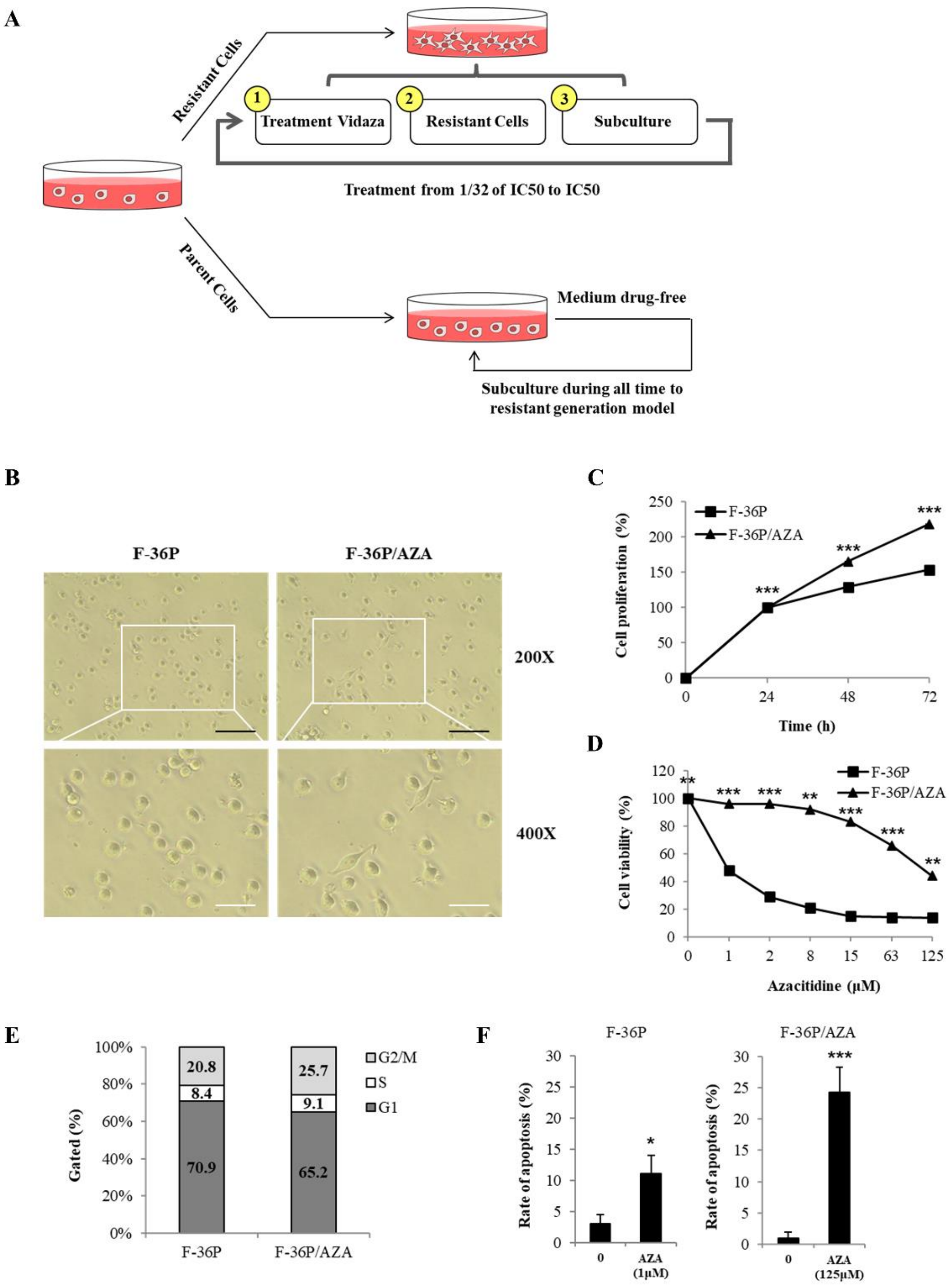

Figure S1. Establishment of azacitidine resistant cell line. (A) A scheme for preparation of azacitidine resistant cell line is shown. (B) Morphological observations of F-36P and F-36P/AZA are shown. Original magnification, ×200 (upper panels), ×400 (lower panels). Black scale bar represents 100 μm; white scale bar, 50 μm. (C) The cell proliferation of F-36P and F-36P/AZA was determined using the WTS assay. (D) The cell viability of the AZA-treated F-36P and F-36P/AZA at 72h was assessed by the WTS assay. F-36P and F-36P/AZA cells were stained with PI and subjected to flow cytometry. Results showed (E) the cell cycle distribution and (F) the percentage of apoptotic cells after treatment of AZA. The IC50 values of F-36P and F-36P/AZA to AZA were 1 μM and 125 μM, respectively. Data are presented as mean ± SD (n = 3), $**p < 0.01$, $***p < 0.001$ vs. Control.

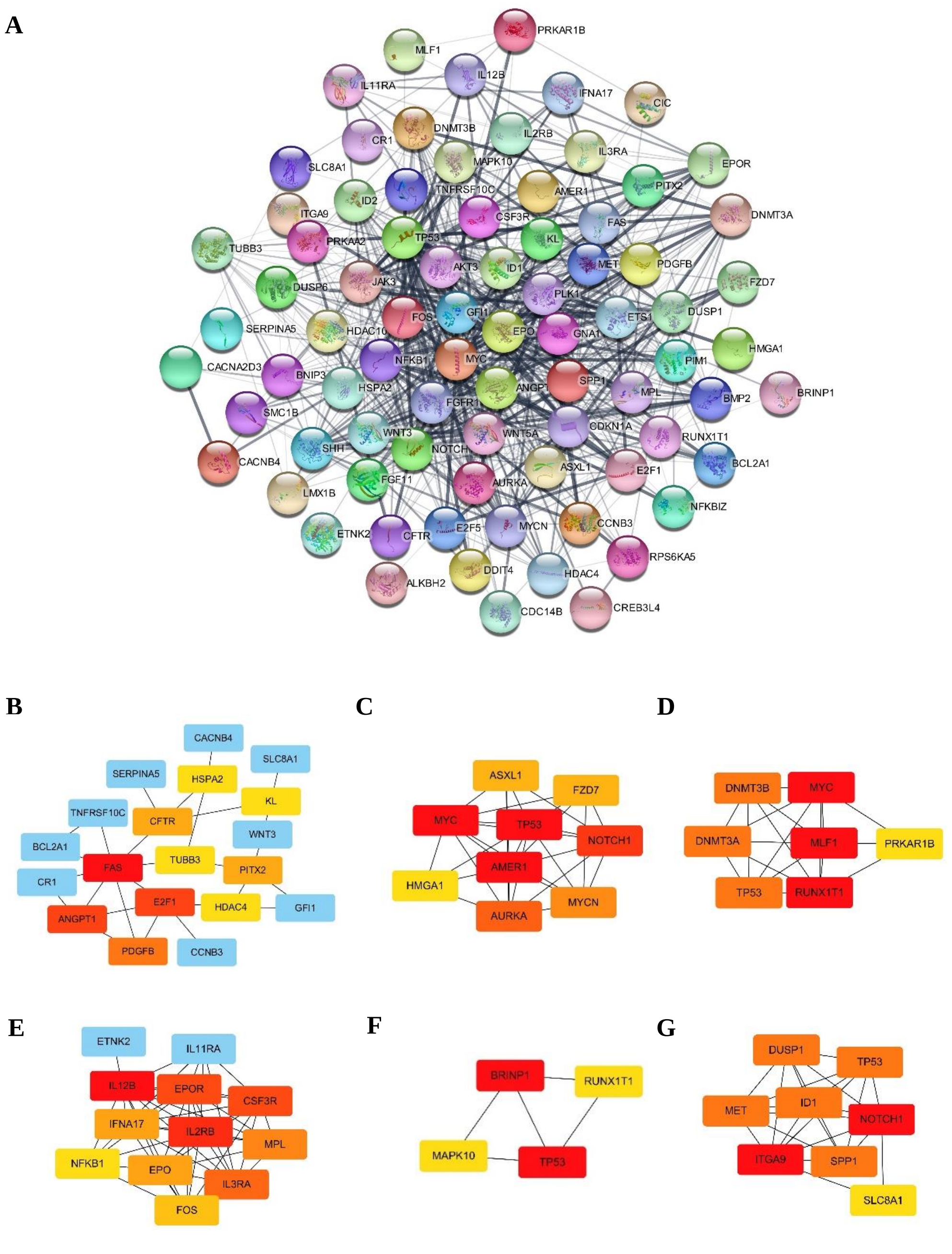

Figure S2. PPI network and subnetworks generated from DEGs. (A) The PPI network was constructed containing 80 DEGs functionally linked with 948 edges using the stringApp of the Cytoscape program (version 3.8.2). The confidence score cut-off was applied with 0.1. Each circle represents a gene (node), and each connection between circles (edge) represent direct or indirect interactions. (B-G) CytoHubba analysis identified the most important module clusters in the PPI network. Genes with the highest number of connections in the network are detected as hub genes. Color depth for ranking of hub genes. Hub genes are indicated in red, and co-expressed genes are indicated in orange, yellow or blue according to their degree of importance. Module 1 (B) contains 19 nodes and 27 edges. Module 2 (C) contains 9 nodes and 29 edges. Module 3 (D) contains 7 nodes and 18 edges. Module 4 (E) contains 12 nodes and 46 edges. Module 5 (F) contains 4 nodes and 5 edges. Module 6 (G) contains 8 nodes and 23 edges.

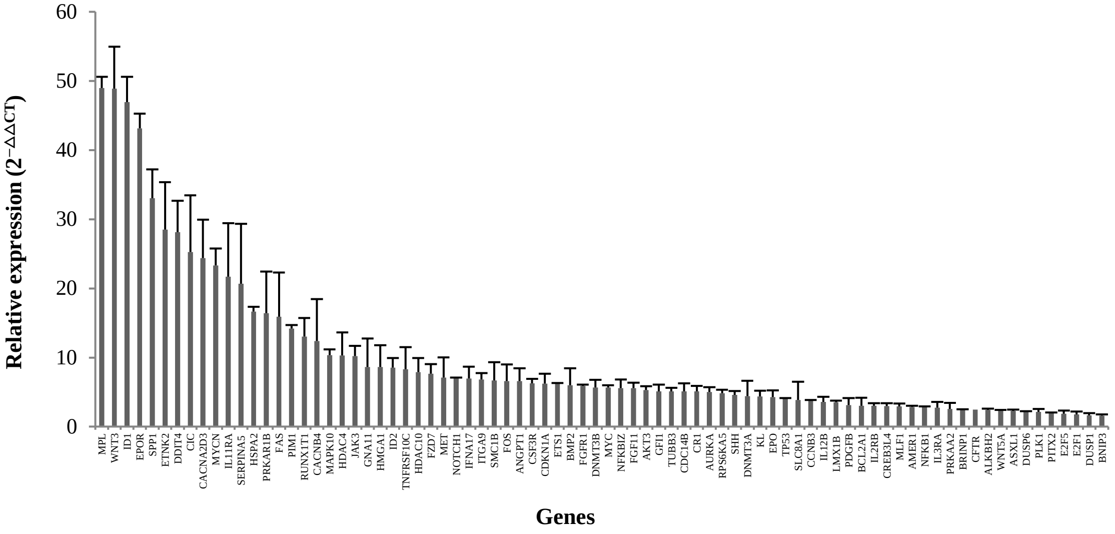

Figure S3. Validation of candidate genes by RT-qPCR. The graph depicts mRNA levels of candidate genes that are differentially expressed between F-36P and F-36P/AZA. All candidate genes were significantly upregulated in F-36P/AZA. The data were used GAPDH as the loading or internal control and analyzed by 2−ΔΔCT method. Differential expression is considered significant at $p<0.05$. Error bars indicate standard deviations (n = 3).

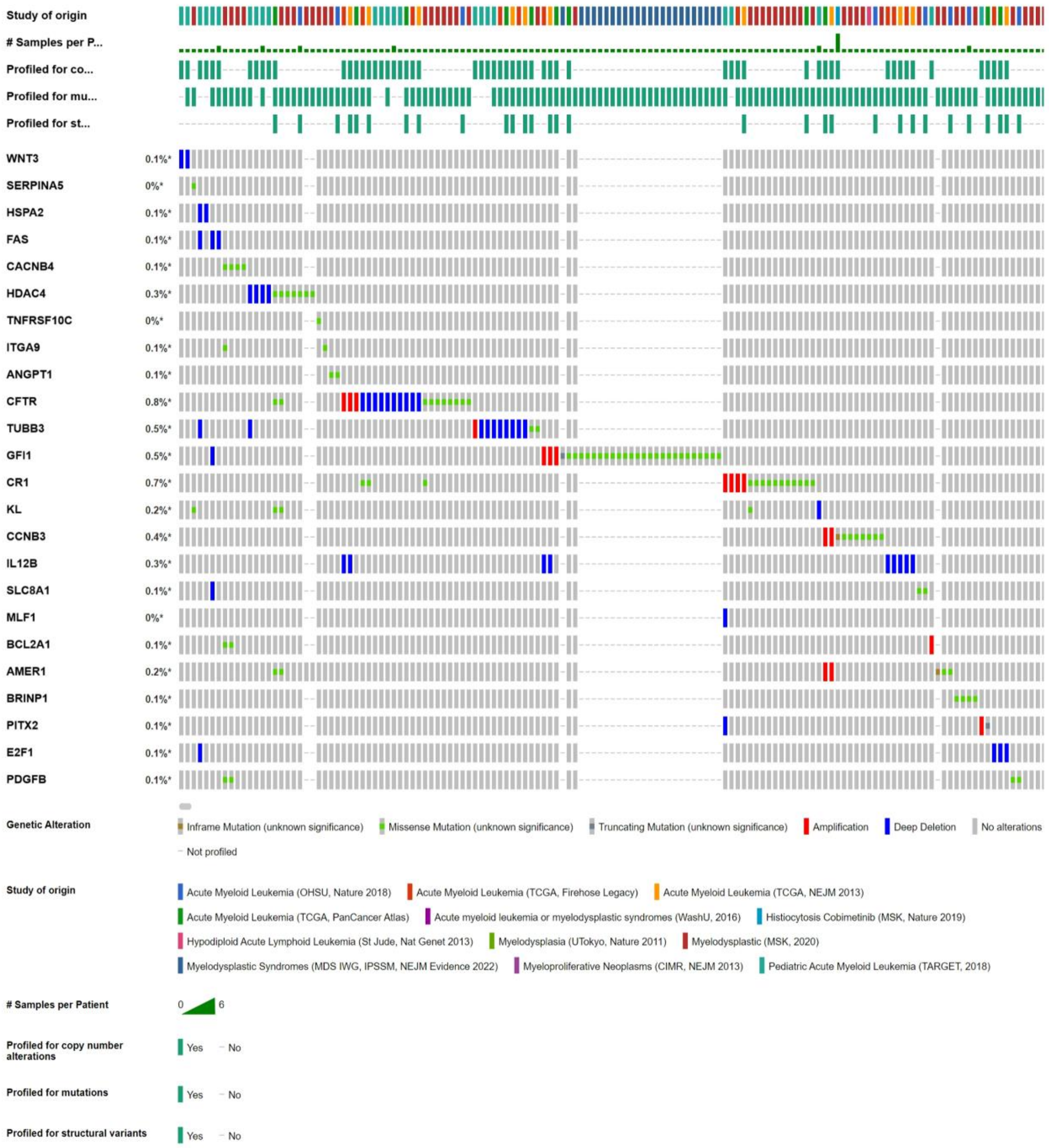

Figure S4. Verification of candidate genes by cBioPortal analysis. Candidate genes were queried for genetic alterations in MDS and AML datasets (http://cbioportal.org/ accessed on 25 January 2023). Alterations were found in 0.1% to 0.8% of the respective analyses and are depicted graphically. Genetic alterations are color coded: Amplification, red; Missense mutations, green; Deep deletion, blue; No alterations, grey. * Not all samples are profiled.

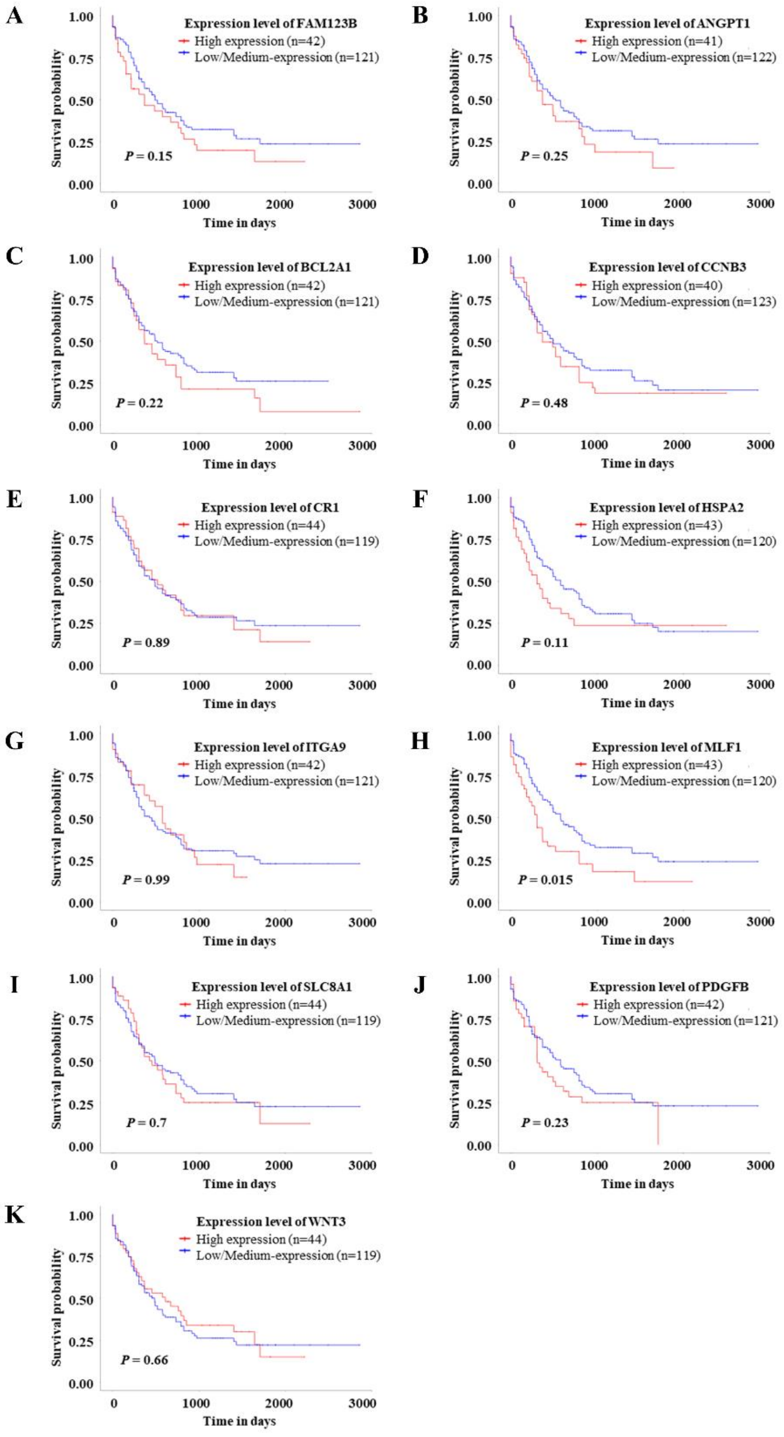

Figure S5. Survival analysis of candidate gene expression in acute myeloid leukemia. (A) FAM123B, (B) ANGPT1, (C) BCL2A1, (D) CCNB3, (E) CR1, (F) HSPA2, (G) ITGA9, (H) MLF1, (I) SLC8A1, (J) PDGFB, (K) WNT3. The results based on the UALCAN database indicated that higher gene expression was associated with poor prognosis in acute myeloid leukemia.

Table S1. Details of primers used for qRT-PCR analysis.

Gene name		Primer Sequences	Accession No.	Product size
AKT3	(F)	TCCAGGCTGTAGCAGACAGA	NM_181690	399
	(R)	AAAACAGCTCGCCCCCATTA		
ALKBH2	(F)	CATCCGGGATCACGTCTCTG	NM_001001655	295
	(R)	AAGACTGTGGTACCAGTGCG		
AMER1	(F)	CCCACACTTGGGAAGCTCAT	NM_152424	464
	(R)	CACAGTGGCATTCCCTTCCT		
ANGPT1	(F)	TTCAGAACCACACGGCTACC	NM_001146.3	406
	(R)	ATCAGCTCCAGTTGCTGCTT		
ASXL1	(F)	AGAAGAAGAAGGAGCGCACG	NM_001164603	238
	(R)	ACCTTGAGCGTGAAAAGGCT		
AURKA	(F)	AGGTGGATCTCTGGAGCCTT	NM_198433	232
	(R)	TTTGCTGTGATCCAGGGGTG		
BCL2A1	(F)	CTGCAGTGCGTCCTACAGAT	NM_004049	293
	(R)	GTATCCACATCCGGGGCAAT		
BMP2	(F)	ATGCAAGCAGGTGGGAAAGT	NM_001200.2	343
	(R)	ATCCAGTCATTCCACCCCAC		
BNIP3	(F)	CAGCATGAGTCTGGACGGAG	NM_004052	435
	(R)	TCAAAAGGTGCTGGTGGAGG		
BRINP1	(F)	TCCTGCAACAAGGGCTACA	NM_014618	155
	(R)	TAGAGGCGTGAGTCCAT		
CACNA2D3	(F)	ATACCGCTCTCCGTGGTGAA	NM_018398.2	322
	(R)	TCCCGTCTTTGTCCCTTTCAT		
CACNB4	(F)	ACACAACCAGTAGCACACCC	NM_001005747.2	314
	(R)	TCCTCGGTTCCTATGGGGTT		
CCNB3	(F)	TACCACCCCAGAGCTCCAAA	NM_033671	230
	(R)	GGCTTCTTTCTTGGGCTGGA		
CDC14B	(F)	CGATCGCCTTTGTTTTGCCA	NM_003671	362
	(R)	TGCAACTTCCATAGGCAGCA		
CDKN1A	(F)	GCGACTGTGATGCGCTAATG	NM_000389	353
	(R)	ATCTGTCATGCTGGTCTGCC		
CFTR	(F)	AAAAGGCCAGCGTTGTCTCC	NM_000492	343
	(R)	ATCGCGATAGAGCGTTCCTC		
CIC	(F)	CTCTGTGGACAACAGGGTCC	NM_015125	419
	(R)	AGCCATGGTCCTGAAAGAGC		
CR1	(F)	CCCACATCCACCCAAGATCC	NM_000573	548
	(R)	TTGTTTGCAGAGTTCGGGGA		
CREB3L4	(F)	GCAGTGACAGTGGCATCTCT	NM_130898	444
	(R)	TGTCCTGAGCTGACTGCTTG		
CSF3R	(F)	GGCCTCAGGGCTATGTGATT	NM_156038.2	509
	(R)	TCTTCCTGCTGGGGTCAAG		
DDIT4	(F)	ACTTCGAGCTGCTCAGTGAC	NM_019058	414
	(R)	TGATGACTCGGAAGCCAGTG		
DNMT1	(F)	ATGAGCAGCCCATCTTCCTG	BC126227	399
	(R)	CCTCATCGTCATCTGCCTCC		

DNMT3A	(F)	CCCAGGCAGCCATTAAGGAA	AF331856.1	553
	(R)	GCGATCATCTCCCTCCTTGG		
DNMT3B	(F)	GGCTGTTTGTCTTGTGGCAG	AF176228	404
	(R)	GGAATGGCAGGGTACAGCTT		
DUSP1	(F)	AAAGGAGGATACGAAGCGTT	NM_004417	500
	(R)	ATAAGGTAAGCAAGGCAGAT		
DUSP6	(F)	AAACAAGGCCCGATCGATGT	NM_005225	350
	(R)	GGTGGGGAAAGGCTGATGAA		
E2F1	(F)	CCAAGTTCGTGTCGCTGC	NM_001951	397
	(R)	GCCTGAATGGCCAAAAGTGT		
E2F5	(F)	ACGACGGGCTGTGCTGAACA	NM_000799.2	301
	(R)	AGGCCGCATCTGGAGGGGA		
EPO	(F)	GACCCCAAGTTCGAGAGCAA	NM_000121	451
	(R)	CCTCGTAGCGGATGTGAGAC		
EPOR	(F)	ATACTATCCACGCCAACGGC	NM_018208	556
	(R)	CGGATCACTGCGTACCTGAG		
ETNK2	(F)	TCCCCTCCCCGGGTAAACTC	NM_001162422	231
	(R)	CCTTATTGAGGTCAGCACGGT		
ETS1	(F)	TGCTGGGCATCTGGACCCTC	NM_000043	259
	(R)	TCTTGGCAGGGCACGCAGTC		
FAS	(F)	CCTCATCCTGCTGTCCAAGG	NM_004112	305
	(R)	AGCGACACTCAGCTGTGAAA		
FGF11	(F)	ACAAGGACAAACCCAACCGT	NM_015850	356
	(R)	GCACTTCTTGGAGGCCAGAT		
FGFR1	(F)	TACTACCACTCACCCGCAGA	NM_005252	474
	(R)	CAGGTTGGCAATCTCGGTCT		
FOS	(F)	CTACCCTACCGCGCCCTAC	NM_003507	361
	(R)	CATGAAGTAGCAGCCCGACA		
FZD7	(F)	GAACGGAGCTCGGAGTTTGA	NM_005263	988
	(R)	CCACCTTCCTCTGGAAACCC		
GFI1	(F)	AGTGCCATCAAGACCCTGTG	NM_002067	547
	(R)	TCTTCAGGATGAACTCCCGC		
GNA11	(F)	TGTGTGTTCAACAACGTGGC	NM_032019	367
	(R)	CTGAGTCAAATCCTGCCGAGA		
HDAC10	(F)	AAACTTCTAGGCTCGCTCGC	NM_006037	311
	(R)	TCCCAGTCCACGATGAGGAT		
HDAC4	(F)	CAGGAAAAGGACGGCACTGA	NM_145904	214
	(R)	GTCTGCCCCTTGGTTTCCTT		
HMGA1	(F)	GAGCTCAATGCCGACCTCTT	NM_021979	407
	(R)	AGTACGCTGCTCTGGTTGTC		
HSPA2	(F)	CTGGACGAGCAGCAGGTAAA	NM_002165	300
	(R)	TCAGCGACACAAGATGCGAT		
ID1	(F)	TGAAAGCCTTCAGTCCCGTG	NM_002166	271
	(R)	TGGTGATGCAGGCTGACAAT		
ID2	(F)	CCCAGGCAGCCATTAAGGAA	AF331856.1	553
	(R)	GCGATCATCTCCCTCCTTGG		
IFNA17	(F)	TCCTGCCTGAAGGACAGACA	NM_021268	342

	(R)	CCAGGCACAAGGGCTGTATT		
IL11RA	(F)<br>(R)	ACCTACATCTGCCAGACCCT<br>TGGTACTGACTCTACCCGCA	NM_147162	420
IL12B	(F)<br>(R)	AAGGGGTGACGTGCGGAGCT<br>GCTGACCTCCACCTGCCGAG	NM_002187	269
IL2RB	(F)<br>(R)	GTGGAGACCCACAGATGCAA<br>CGGAATGGTGTCCTTCCCAA	NM_000878.2	300
IL3RA	(F)<br>(R)	ACGCCGACTATTCTATGCCG<br>CTCGTACTGTTGACGCCTGT	NM_002183	287
ITGA9	(F)<br>(R)	GACCGCGATGATGAGTGGAT<br>CATCAATGGTGGACGGGTGA	NM_002207	436
JAK3	(F)<br>(R)	CTGTCTGTGTCCAGAACCCC<br>AGCCCCGGTAAATCTTGGTG	NM_000215	375
KL	(F)<br>(R)	GTGGCCGAGAGAGTTTTGGA<br>GGGAGGTCTCCGTACTTGAA	AB005142.1	359
LMX1B	(F)<br>(R)	AGAGACACTGGCAGCTGA<br>TCGTTCCCTGGCATTTGGG	NM_002316	291
MAPK10	(F)<br>(R)	TTCACACCCCAGAAAACGCT<br>CTTGGGCCGATTCTCCACAT	NM_002753	522
MET	(F)<br>(R)	CGCACAAAGCAAGCCAGATT<br>GAGGCATTGACTGCAGGACT	NM_000245	553
MLF1	(F)<br>(R)	TCCATTCTTGCACACCGAGA<br>TGGAGCTCGACGAGTTTGAG	NM_022443	351
MPL	(F)<br>(R)	AGCTGCCTCATCTCAGGACT<br>AAGTGGCAGCGAGAGAACTG	NM_005373.2	344
MYC	(F)<br>(R)	TCGGTGCAGCCGTATTTCTA<br>TGATGAAGGTCTCGTCGTCC	AH002905	325
MYCN	(F)<br>(R)	TTCGGTCCAGCTTTCTCACG<br>CAAGTCCGAGCGTGTTCAAT	NM_005378.4	202
NFKB1	(F)<br>(R)	GGGCACTGGAAGTACAGGTC<br>TCCTGCACAGCAGTGAGATG	NM_003998.2	435
NFKBIZ	(F)<br>(R)	TCGTTGTCTGATGGACCTGC<br>TTGGTTCATAAGCGGGGGAC	NM_001005474	466
NOTCH1	(F)<br>(R)	TGCACACTATTCTGCCCCAG<br>ACTTGAAGGCCTCCGGAATG	NM_017617	309
PDGFB	(F)<br>(R)	ATGATCGCCGAGTGCAAGAC<br>CGTGTGCTTGAATTTCCGGT	NM_002608.4	411
PIM1	(F)<br>(R)	TTCATCACGGAAAGGGGAGC<br>GGCCCCTGATGATCTCTTCG	NM_002648	361
PITX2	(F)<br>(R)	TCGGCGTGTGTGCAATTAGAG<br>CGGCCCAGTTGTTGTAGGAA	NM_153427.2	376
PLK1	(F)<br>(R)	TGACTCAACACGCCTCATCC<br>AGGAGACTCAGGCGGTATGT	NM_005030	390
PRKAA2	(F)<br>(R)	TCTTCTTTGTGGCACCCTCC<br>ATAAGCCACTGCAAGCTGGT	NM_006252	355
PRKAR1B	(F)<br>(R)	AAGCCAAGACGGACCTCAAG<br>ACGTACTCCTCATTGGGGGA	NM_001164759.1	292

RPS6KA5	(F)	CAGCTCCTCACTGTCAAGCA	NM_004755	425
	(R)	AGGGCAAGCACAATCTCTCC		
RUNX1T1	(F)	TGCAGCAGTTTGGCAATGAC	NM_004349	348
	(R)	TGGTTCTGTCTGGAGTTCGC		
SERPINA5	(F)	AATGCCCTTTTCACCGACCT	NM_000624	482
	(R)	AGCCACTTCCTCAGCGTTTT		
SHH	(F)	TCCACTGCTCGGTGAAAGCAG	NM_000193	674
	(R)	AGCTGTGCTCCTCGATGACC		
SLC8A1	(F)	GCAAGCAGAGGGGGATGATT	NM_001112800.1	503
	(R)	GGTAAGGGCCACAGTACCAC		
SMC1B	(F)	AGTTTTCGTCCTGCCCCATT	NM_148674.3	273
	(R)	TCCGTGTCTCTTGCTGCTTT		
SPP1	(F)	GAATCTCCTAGCCCCACAGACC	NM_000582	378
	(R)	TGTGAGGTGATGTCCTCGTCT		
TNFRSF10C	(F)	ACAGAGGCACAGCTTCAAGG	NM_003841.2	355
	(R)	GGTTTCCACAGTGGCATTGG		
TP53	(F)	TGGGACAGCCAAGTCTGTGA	AB082923	436
	(R)	ACTGGAGTCTTCCAGTGTGA		
TUBB3	(F)	TCACTACACGGAGGGGGCGG	NM_006086.3	418
	(R)	CGGGAAGCGCAAGGAGGTGG		
WNT3	(F)	AACAAGCACAACAACGAGGC	NM_030753	427
	(R)	TTTCCTTCCGCTTCTCCGTC		
WNT5A	(F)	CTTTGGGGATGGCTGGAAGT	NM_003392	352
	(R)	ATCTGCATCACCCTGCCAAA		
GAPDH	(F)	AAGGACTCATGACCACAGTC	NM_001289745.2	160
	(R)	TTCAGCTCAGGGATGACCTT		

Table S2. Details of primers used for pyrosequencing.

Gene name		Primer Sequences	Position	Accession No.	Product size
AMER1	(F)	GTTTTTTTGGGGTTTAGAGAGAGTTGG	17171-17417	NG021345.1	247
	(R)	CCTCAAACTTCCAAACACTATTATAACATT			
ANGPT1	(F)	GTTTAAGGTAATTGTTTGTGGAAAG	4883-5073	NG_029405.1	191
	(R)	TACACATCCAAACCAAACTAAAAAC			
BCL2A1	(F)	GATGGGGTTTTATTGTGTTAGTTAG	3678-3804	NG_029487.1	127
	(R)	AAAATACCCTCTTTAAACCAAACAC			
CACNB4	(F)	TAGTTTTTTGTTGGGTAGGATGTTT	129991-130180	NG_012641.1	190
	(R)	TCCAAACCTAACATTTTATCTACCTTC			
CCNB3	(F)	TTGGATTATAGTTTTTTTTGATTGAA	3379-3667	NG_012880.1	289
	(R)	CACAATACCTACCATCCTCTATAAAC			
CFTR	(F)	AGGAGGAGGAGGAAGGTAGG	18707-18909	NG_016465.4	203
	(R)	CCCAACACCAAACCCATCC			
CR1	(F)	GGGAAGTTGTGGTTAAAAGTA	4980-5142	NG_007481.1	163
	(R)	ATTCTCCCCAAACACATCTA			
DBC1	(F)	TGGGGGTATTTGGTTTTTAGTAGT	139562-139837	AL138894.11	276
	(R)	CACTTAAACTTCCCTCTCCATT			
E2F1	(F)	TGGGTTTTTATTATGTTGATTAGGTT	4118-4326	NG_046988.1	209
	(R)	ACTTACTCTATACCCCATCCTCAATT			
FAS	(F)	TGAATTAATGGAGTTTTTTTTAATT	5026-5154	NG_009089.2	129
	(R)	TCTTTAAACACCTATATATCACTCTTAC			
GFI1	(F)	TGTTGTGGAAATATTTGGGGATAA	62924153 - 62924273	NM_005263.2	121
	(R)	CCACCTCTTCCAAATTTAACCAT			
HDAC4	(F)	ATTTGTGGGTTATTTGGTTTATGAG	53066-53244	NG_009235.1	179
	(R)	ACAATATCCACTCCTTTAAATAATTC			
HSPA2	(F)	GGAAAATTAGGGAGATGAATTT	139063-139201	AL049869.6	139
	(R)	ATTAAATCTAAAAATTCCCAAAAAC			
IL12B	(F)	TAGATTTTTTGGGTTTTATA	3567357-3567524	NM_002187.2	168
	(R)	TACTAAACCCTTTACCCTTC			
ITGA9	(F)	ATTTAGTAGGGTTGAGTGGGATTT	4205-4447	NG_016166.1	243
	(R)	TCCTCTACTCCTTCACCCAATTATA			
Klotho	(F)	AGTGAGAGTAGGTGTTTTTTTAG	4545-4700	NG_011485.1	156
	(R)	CAAATTCATTCTCTTTACCTACC			
MLF1	(F)	GGATATTGGGTGTTATTTGGTTTAA	4679-4785	NG_027720.1	107
	(R)	CCCTTTCTATAACCTTAAACATACC			
NCX1	(F)	TGTTATATGAGTTAATTTAAATGGA	14033-14228	AC007281.3	196
	(R)	AATAAACAAACCAAAACCTAATTAT			
PDGFB	(F)	TATTTAGGGAATGAAAAATGGG	4903-5092	NG_012111.1	190
	(R)	AAAACTATCTCCACCCACCTCT			
PITX2	(F)	TGGAAGAGAAGAAAAAGTAA	4783-4967	NG_007120.1	185
	(R)	TCCCAAACAACCCCATAAAC			
SERPINA5	(F)	TGGTTTAGTTGGTGGTGTAA	24779-24907	NG_032908.2	129
	(R)	AACAACCCCTTCCTTTCCTA			
TNFRSF10C	(F)	TGTTTGGAAGTGATTGTTGTAAGTG	5023-5211	NG_032578.1	189
	(R)	AAACCCTCCTTCACCCCTTACATCTCT			

TUBB3	(F)<br>(R)	GGTTTTTGTTTGGTTTGTG<br>TCCTCCACCAAAAAACTACCAT	5560-5695	NG_027810.1	136
WNT3	(F)<br>(R)	TGATTTTTGATAAAAGATTTAAATG<br>AAAATATTTCCAAAAACCCC	4345-4583	NG_008084.2	239

Table S3. GO analysis of AZA resistance-related DEGs.

Category	Term	Description	Count	*P*-Value
BP	GO:0043066	Negative regulation of apoptotic process	17	2.92.E-10
	GO:0000122	Negative regulation of transcription from RNA polymerase II promoter	20	6.39E-10
	GO:0045944	Positive regulation of transcription from RNA polymerase II promoter	20	9.64E-08
	GO:0000165	MAPK cascade	9	3.16.E-05
	GO:0007050	Cell cycle arrest	7	5.29.E-05
	GO:0042493	Response to drug	8	5.66.E-04
	GO:0006355	Regulation of transcription, DNA-templated	15	9.90.E-03
	GO:0016055	Wnt signaling pathway	5	1.17.E-02
	GO:0030097	Hemopoiesis	3	3.15.E-02
	GO:0050671	Positive regulation of lymphocyte proliferation	2	3.25.E-02
CC	GO:0005654	Nucleoplasm	29	7.87.E-06
	GO:0009986	Cell surface	12	2.11.E-05
	GO:0005576	Extracellular region	18	4.50.E-04
	GO:0005634	Nucleus	38	9.60.E-04
	GO:0043234	Protein complex	8	2.05.E-03
	GO:0005829	Cytosol	25	5.78.E-03
	GO:0005886	Plasma membrane	28	1.25.E-02
	GO:0005615	Extracellular space	12	3.03.E-02
	GO:0005887	Integral component of plasma membrane	12	4.12.E-02
	GO:0005737	Cytoplasm	31	4.61.E-02
MF	GO:0008134	Transcription factor binding	12	8.26.E-08
	GO:0004896	Cytokine receptor activity	6	6.64.E-07
	GO:0003700	Transcription factor activity, sequence-specific DNA binding	16	3.35.E-05
	GO:0046982	Protein heterodimerization activity	9	1.47.E-03
	GO:0042802	Identical protein binding	11	2.52.E-03
	GO:0044212	Transcription regulatory region DNA binding	6	3.23.E-03
	GO:0051718	DNA (cytosine-5-)-methyltransferase activity, acting on CpG substrates	2	9.34.E-03
	GO:0004888	Transmembrane signaling receptor activity	5	1.80.E-02
	GO:0019899	Enzyme binding	6	1.99.E-02
	GO:0019901	Protein kinase binding	6	3.15.E-02

Table S4. The 23 most differentially expressed genes from the volcano plot.

No.	Gene Name	Log_2 fold change	P-value	Description
1	MPL	3.86	0.002	MPL proto-oncogene, thrombopoietin receptor
2	RIN1	2.80	0.048	Ras interaction/interference protein 1
3	CCNB3	2.44	0.033	G2/mitotic-specific cyclin-B3
4	PRKAR1B	2.30	0.031	cAMP-dependent protein kinase type I-beta regulatory subunit
5	AKT3	2.19	0.011	V-akt murine thymoma viral oncogene homolog 3
6	JAK3	1.98	0.013	Tyrosine-protein kinase JAK3
7	CSF3R	1.66	0.011	Colony stimulating factor 3 receptor (granulocyte)
8	CD19	1.61	0.009	B-lymphocyte surface antigen B4
9	PIK3R5	1.28	0.035	Phosphatidylinositol 4,5-bisphosphate 3-kinase regulatory subunit
10	NTHL1	1.01	0.049	Bifunctional DNA N-glycosylase/DNA-(apurinic or apyrimidinic site) lyase
11	PRKAR2B	-1.04	0.024	cAMP-dependent protein kinase type II-beta regulatory subunit
12	FGF12	-1.12	0.032	Fibroblast growth factor homologous factor 1
13	PLA2G4A	-1.20	0.010	Phospholipase A2, group IVA (cytosolic, calcium-dependent)
14	DNMT1	-1.23	0.033	DNA (cytosine-5-)-methyltransferase 1
15	PPP2R2C	-1.32	0.038	Serine/threonine-protein phosphatase 2A 55 kDa regulatory subunit B gamma isoform
16	CDKN2D	-1.36	0.002	Cyclin-dependent kinase inhibitor 2D (p19, inhibits CDK4)
17	CCND2	-1.50	0.036	G1/S-specific cyclin-D2
18	FST	-1.58	0.009	Activin-binding protein
19	FGFR3	-1.65	0.100	Fibroblast growth factor receptor 3
20	RELN	-1.70	0.029	Reelin
21	WNT5B	-2.11	0.015	Wingless-type MMTV integration site family, member 5B
22	THBS1	-2.62	0.040	Thrombospondin 1
23	LEPR	-2.67	0.004	Leptin receptor

Table S5. DEGs between F-36P and F-36P/AZA cells.

	Gene Name	Accession Number	Description
1	AKT3	NM_181690.1	V-akt murine thymoma viral oncogene homolog 3
2	ALKBH2	NM_001001655.2	Alpha-ketoglutarate-dependent dioxygenase alkB homolog 2
3	AMER1	NM_152424.3	Wilms tumor gene on the X chromosome protein
4	ANGPT1	NM_001146.3	Angiopoietin 1
5	ASXL1	NM_001164603.1	Additional sex combs like transcriptional regulator 1
6	AURKA	NM_001323303.2	Serine/threonine-protein kinase aurora-A
7	BCL2A1	NM_004049.2	Hemopoietic-specific early response protein
8	BMP2	NM_001200.2	Bone morphogenetic protein 2A
9	BNIP3	NM_004052.2	BCL2/adenovirus E1B 19 kDa protein-interacting protein 3
10	BRINP1	NM_014618	Bone morphogenetic protein/retinoic acid inducible neural-specific 1
11	CACNA2D3	NM_018398.2	Calcium channel, voltage-dependent, alpha 2/delta subunit 3
12	CACNB4	NM_001005747.2	Voltage-dependent L-type calcium channel subunit beta-4
13	CCNB3	NM_033671.1	G2/mitotic-specific cyclin-B3
14	CDC14B	NM_003671.3	Dual specificity protein phosphatase CDC14B
15	CDKN1A	NM_000389.2	Cyclin-dependent kinase inhibitor 1A (p21, Cip1)
16	CFTR	NM_000492	Cystic fibrosis transmembrane conductance regulator (ATP-binding cassette sub-family C, member 7)
17	CIC	NM_015125.3	Capicua transcriptional repressor
18	CR1	NM_000573	Complement component (3b/4b) receptor 1 (Knops blood group)
19	CREB3L4	NM_130898.2	Cyclic AMP-responsive element-binding protein 3-like protein 4
20	CSF3R	NM_156038.2	Colony stimulating factor 3 receptor (granulocyte)
21	DDIT4	NM_019058.2	Protein regulated in development and DNA damage response 1
22	DNMT3A	NM_022552.3	DNA (cytosine-5-)-methyltransferase 3 alpha
23	DNMT3B	AF176228	DNA (cytosine-5-)-methyltransferase 3 beta
24	DUSP1	NM_004417	Mitogen-activated protein kinase phosphatase 1
25	DUSP6	NM_001946.2	Mitogen-activated protein kinase phosphatase 3
26	E2F1	NM_005225.1	Retinoblastoma-associated protein 1
27	E2F5	NM_001951.3	E2F transcription factor 5, p130-binding
28	EPO	NM_000799.2	Erythropoietin
29	EPOR	NM_000121.2	Erythropoietin receptor
30	ETNK2	NM_018208	Ethanolamine kinase-like protein
31	ETS1	NM_001162422	V-ets avian erythroblastosis virus E26 oncogene homolog 1
32	FAS	NM_152876.1	Tumor necrosis factor receptor superfamily member 6

33	FGF11	NM_004112.2	Fibroblast growth factor homologous factor 3
34	FGFR1	NM_015850.2	Basic fibroblast growth factor receptor 1
35	FOS	NM_005252.2	FBJ murine osteosarcoma viral oncogene homolog
36	FZD7	NM_003507.1	Frizzled class receptor 7
37	GFI1	NM_005263	Growth factor independent 1 transcription repressor
38	GNA11	NM_002067.1	Guanine nucleotide binding protein (G protein), alpha 11 (Gq class)
39	HDAC10	NM_032019.5	Histone deacetylase 10
40	HDAC4	NM_006037.3	Histone deacetylase 4
41	HMGA1	NM_145904.1	High mobility group protein HMG-I/HMG-Y
42	HSPA2	NM_021979.3	Heat shock-related 70 kDa protein 2
43	ID1	NM_002165.2	Inhibitor of DNA binding 1, dominant negative helix-loop-helix protein
44	ID2	NM_002166.4	Inhibitor of DNA binding 2, dominant negative helix-loop-helix protein
45	IFNA17	NM_021268.2	Interferon, alpha 17
46	IL11RA	NM_147162.1	Interleukin-11 receptor subunit alpha
47	IL12B	NM_002187.2	Cytotoxic lymphocyte maturation factor 40 kDa subunit
48	IL2RB	NM_000878.2	High affinity IL-2 receptor subunit beta
49	IL3RA	NM_002183.2	Interleukin 3 receptor, alpha (low affinity)
50	ITGA9	NM_002207.2	Integrin alpha-RLC
51	JAK3	NM_000215.2	Tyrosine-protein kinase JAK3
52	KL	AB005142.1	Klotho
53	LMX1B	NM_002316	LIM homeobox transcription factor 1, beta
54	MAPK10	NM_002753.2	Stress-activated protein kinase JNK3
55	MET	NM_000245.2	MET proto-oncogene, receptor tyrosine kinase
56	MLF1	NM_022443.3	Myelodysplasia-myeloid leukemia factor 1
57	MPL	NM_005373.2	MPL proto-oncogene, thrombopoietin receptor
58	MYC	NM_002467.3	V-myc avian myelocytomatosis viral oncogene homolog
59	MYCN	NM_005378.4	V-myc avian myelocytomatosis viral oncogene neuroblastoma derived homolog
60	NFKB1	NM_003998.2	Nuclear factor of kappa light polypeptide gene enhancer in B-cells 1
61	NFKBIZ	NM_001005474.1	Nuclear factor of kappa light polypeptide gene enhancer in B-cells inhibitor, zeta
62	NOTCH1	NM_017617.3	Translocation-associated notch protein TAN-1
63	PDGFB	NM_033016.2	Platelet-derived growth factor beta polypeptide
64	PIM1	NM_002648.2	Pim-1 proto-oncogene, serine/threonine kinase
65	PITX2	NM_000325.5	RIEG bicoid-related homeobox transcription factor

66	PLK1	NM_005030	Serine/threonine-protein kinase PLK1
67	PRKAA2	NM_006252.2	5'-AMP-activated protein kinase catalytic subunit alpha-2
68	PRKAR1B	NM_001164759.1	cAMP-dependent protein kinase type I-beta regulatory subunit
69	RPS6KA5	NM_004755.2	Nuclear mitogen- and stress-activated protein kinase 1
70	RUNX1T1	NM_004349.2	Runt-related transcription factor 1
71	SERPINA5	NM_000624	Serpin peptidase inhibitor, clade A (alpha-1 antiproteinase, antitrypsin), member 5
72	SHH	NM_000193	Shh unprocessed N-terminal signaling and C-terminal autoprocessing domains
73	SLC8A1	NM_001112800.1	Solute carrier family 8 (sodium/calcium exchanger), member 1
74	SMC1B	NM_148674.3	Structural maintenance of chromosomes protein 1B
75	SPP1	NM_000582.2	Secreted phosphoprotein 1
76	TNFRSF10C	NM_003841.2	Tumor necrosis factor receptor superfamily, member 10c, decoy without an intracellular domain
77	TP53	NM_000546.2	Cellular tumor antigen p53
78	TUBB3	NM_006086.3	Tubulin, beta 3 class III
79	WNT3	NM_030753.3	Wingless-type MMTV integration site family, member 3
80	WNT5A	NM_003392.3	Wingless-type MMTV integration site family, member 5A

Table S6. Functional roles of module genes.

No.	Gene symbol	Full name	Function
1	WNT3	Wnt Family Member 3	Wingless-type MMTV integration site family, member 3; Ligand for members of the frizzled family of seven transmembrane receptors. Wnt-3 and Wnt-3a play distinct roles in cell-cell signaling during morphogenesis of the developing neural tube (By similarity).
2	SERPINA5	Serpin Family A Member 5	Serpin peptidase inhibitor, clade A (alpha-1 antiproteinase, antitrypsin), member 5; Heparin-dependent serine protease inhibitor acting in body fluids and secretions. Inactivates serine proteases by binding irreversibly to their serine activation site. Involved in the regulation of intravascular and extravascular proteolytic activities. Plays hemostatic roles in the blood plasma. Acts as a procoagulant and proinflammatory factor by inhibiting the anticoagulant activated protein C factor as well as the generation of activated protein C factor by the thrombin/thrombomodulin complex. Acts as an anticoagulant factor by inhibiting blood coagulation factors like prothrombin, factor XI, factor Xa, plasma kallikrein and fibrinolytic enzymes such as tissue- and urinary- type plasminogen activators. In seminal plasma, inactivates several serine proteases implicated in the reproductive system. Inhibits the serpin acrosin; indirectly protects component of the male genital tract from being degraded by excessive released acrosin. Inhibits tissue-and urinary-type plasminogen activator, prostate-specific antigen and kallikrein activities; has a control on the sperm motility and fertilization. Inhibits the activated protein C-catalyzed degradation of SEMG1 and SEMG2; regulates the degradation of semenogelin during the process of transfer of spermatozoa from the male reproductive tract into the female tract. In urine, inhibits urinary-type plasminogen activator and kallikrein activities. Inactivates membrane-anchored serine proteases activities such as MPRSS7 and TMPRSS11E. Inhibits urinary-type plasminogen activator-dependent tumor cell invasion and metastasis. May also play a non-inhibitory role in seminal plasma and urine as a hydrophobic hormone carrier by its binding to retinoic acid.
3	HSPA2	Heat Shock Protein Family A (Hsp70) Member 2	Heat shock-related 70 kDa protein 2; Molecular chaperone implicated in a wide variety of cellular processes, including protection of the proteome from stress, folding and transport of newly synthesized polypeptides, activation of proteolysis of misfolded proteins and the formation and dissociation of protein complexes. Plays a pivotal role in the protein quality control system, ensuring the correct folding of proteins, the re-folding of misfolded proteins and controlling the targeting of proteins for subsequent degradation. This is achieved through cycles of ATP binding, ATP hydrolysis and ADP release, mediated by co-chaperones. The affinity for polypeptides is regulated by its nucleotide bound state. In the ATP-bound form, it has a low affinity for substrate proteins. However, upon hydrolysis of the ATP to ADP, it undergoes a conformational change that increases its affinity for substrate proteins. It goes through repeated cycles of ATP hydrolysis and nucleotide exchange, which permits cycles of substrate binding and release. Plays a role in spermatogenesis. In association with SHCBP1L may participate in the maintenance of spindle integrity during meiosis in male germ cells (By similarity); Heat shock 70kDa proteins
4	FAS	Fas Cell Surface Death Receptor	Tumor necrosis factor receptor superfamily member 6; Receptor for TNFSF6/FASLG. The adapter molecule FADD recruits caspase-8 to the activated receptor. The resulting death- inducing

			signaling complex (DISC) performs caspase-8 proteolytic activation which initiates the subsequent cascade of caspases (aspartate-specific cysteine proteases) mediating apoptosis. FAS-mediated apoptosis may have a role in the induction of peripheral tolerance, in the antigen-stimulated suicide of mature T-cells, or both. The secreted isoforms 2 to 6 block apoptosis (in vitro); CD molecules
5	CACNB4	Calcium Voltage-Gated Channel Auxiliary Subunit Beta 4	Voltage-dependent L-type calcium channel subunit beta-4; The beta subunit of voltage-dependent calcium channels contributes to the function of the calcium channel by increasing peak calcium current, shifting the voltage dependencies of activation and inactivation, modulating G protein inhibition and controlling the alpha-1 subunit membrane targeting.
6	HDAC4	Histone Deacetylase 4	Histone deacetylase 4; Responsible for the deacetylation of lysine residues on the N-terminal part of the core histones (H2A, H2B, H3 and H4). Histone deacetylation gives a tag for epigenetic repression and plays an important role in transcriptional regulation, cell cycle progression and developmental events. Histone deacetylases act via the formation of large multiprotein complexes. Involved in muscle maturation via its interaction with the myocyte enhancer factors such as MEF2A, MEF2C and MEF2D. Involved in the MTA1-mediated epigenetic regulation of ESR1 expression in breast cancer. Deacetylates HSPA1A and HSPA1B at 'Lys-77' leading to their preferential binding to co-chaperone STUB1.
7	TNFRSF10C	TNF Receptor Superfamily Member 10c	Tumor necrosis factor receptor superfamily, member 10c, decoy without an intracellular domain; Receptor for the cytotoxic ligand TRAIL. Lacks a cytoplasmic death domain and hence is not capable of inducing apoptosis. May protect cells against TRAIL mediated apoptosis by competing with TRAIL-R1 and R2 for binding to the ligand; CD molecules
8	ITGA9	Integrin Subunit Alpha 9	Integrin alpha-RLC; Integrin alpha-9/beta-1 (ITGA9:ITGB1) is a receptor for VCAM1, cytotactin and osteopontin. It recognizes the sequence A-E- I-D-G-I-E-L in cytotactin.
9	ANGPT1	Angiopoietin 1	Angiopoietin 1; Binds and activates TEK/TIE2 receptor by inducing its dimerization and tyrosine phosphorylation. Plays an important role in the regulation of angiogenesis, endothelial cell survival, proliferation, migration, adhesion and cell spreading, reorganization of the actin cytoskeleton, but also maintenance of vascular quiescence. Required for normal angiogenesis and heart development during embryogenesis. After birth, activates or inhibits angiogenesis, depending on the context. Inhibits angiogenesis and promotes vascular stability in quiescent vessels, where endothelial cells have tight contacts. In quiescent vessels, ANGPT1 oligomers recruit TEK to cell-cell contacts, forming complexes with TEK molecules from adjoining cells, and this leads to preferential activation of phosphatidylinositol 3-kinase and the AKT1 signaling cascades. In migrating endothelial cells that lack cell-cell adhesions, ANGT1 recruits TEK to contacts with the extracellular matrix, leading to the formation of focal adhesion complexes, activation of PTK2/FAK and of the downstream kinases MAPK1/ERK2 and MAPK3/ERK1, and ultimately to the stimulation of sprouting angiogenesis. Mediates blood vessel maturation/stability. Implicated in endothelial developmental processes later and distinct from that of VEGF. Appears to play a crucial role in mediating reciprocal interactions between the endothelium and surrounding matrix and mesenchyme; Fibrinogen C domain containing
10	CFTR	CF Transmembrane Conductance Regulator	Cystic fibrosis transmembrane conductance regulator (ATP-binding cassette sub-family C, member 7); Epithelial ion channel that plays an important role in the regulation of epithelial ion and water transport and fluid homeostasis. Mediates the transport of chloride ions across the cell membrane. Channel activity is

			coupled to ATP hydrolysis. The ion channel is also permeable to HCO(3-); selectivity depends on the extracellular chloride concentration. Exerts its function also by modulating the activity of other ion channels and transporters. Plays an important role in airway fluid homeostasis. Contributes to the regulation of the pH and the ion content of the airway surface fluid layer and thereby plays an important role in defense against pathogens. Modulates the activity of the epithelial sodium channel (ENaC) complex, in part by regulating the cell surface expression of the ENaC complex. Inhibits the activity of the ENaC channel containing subunits SCNN1A, SCNN1B and SCNN1G. Inhibits the activity of the ENaC channel containing subunits SCNN1D, SCNN1B and SCNN1G, but not of the ENaC channel containing subunits SCNN1A, SCNN1B and SCNN1G. May regulate bicarbonate secretion and salvage in epithelial cells by regulating the transporter SLC4A7. Can inhibit the chloride channel activity of ANO1. Plays a role in the chloride and bicarbonate homeostasis during sperm epididymal maturation and capacitation; ATP binding cassette subfamily C
11	TUBB3	Tubulin Beta 3 Class III	Tubulin, beta 3 class III; Tubulin is the major constituent of microtubules. It binds two moles of GTP, one at an exchangeable site on the beta chain and one at a non-exchangeable site on the alpha chain. TUBB3 plays a critical role in proper axon guidance and mantainance; Belongs to the tubulin family.
12	GFI1	Growth Factor Independent 1 Transcriptional Repressor	Growth factor independent 1 transcription repressor; Transcription repressor essential for hematopoiesis. Functions in a cell-context and development-specific manner. Binds to 5'-TAAATCAC[AT]GCA-3' in the promoter region of a large number of genes. Component of several complexes, including the EHMT2-GFI1-HDAC1, AJUBA-GFI1-HDAC1 and RCOR-GFI-KDM1A-HDAC complexes, that suppress, via histone deacetylase (HDAC) recruitment, a number of genes implicated in multilineage blood cell development. Regulates neutrophil differentiation, promotes proliferation of lymphoid cells, and is required for granulocyte development. Mediates, together with U2AF1L4, the alternative splicing of CD45 and controls T-cell receptor signaling. Regulates the endotoxin- mediated Toll-like receptor (TLR) inflammatory response by antagonizing RELA. Cooperates with CBFA2T2 to regulate ITGB1- dependent neurite growth. Controls cell-cycle progression by repressing CDKNIA/p21 transcription in response to TGFB1 via recruitment of GFI1 by ZBTB17 to the CDKNIA/p21 and CDKNIB promoters. Required for the maintenance of inner ear hair cells; SNAG transcriptional repressors
13	CR1	Complement C3b/C4b Receptor 1 (Knops Blood Group)	Complement component (3b/4b) receptor 1 (Knops blood group); Mediates cellular binding of particles and immune complexes that have activated complement; Blood group antigens
14	KL	Klotho	Klotho; May have weak glycosidase activity towards glucuronylated steroids. However, it lacks essential active site Glu residues at positions 239 and 872, suggesting it may be inactive as a glycosidase in vivo. May be involved in the regulation of calcium and phosphorus homeostasis by inhibiting the synthesis of active vitamin D (By similarity). Essential factor for the specific interaction between FGF23 and FGFR1 (By similarity); Belongs to the glycosyl hydrolase 1 family. Klotho subfamily.
15	CCNB3	Cyclin B3	G2/mitotic-specific cyclin-B3; Cyclins are positive regulatory subunits of the cyclin- dependent kinases (CDKs), and thereby play an essential role in the control of the cell cycle, notably via their destruction during cell division. Its tissue specificity suggest that it may be required during early meiotic prophase I.

16	IL12B	Interleukin 12B	Cytotoxic lymphocyte maturation factor 40 kDa subunit; Cytokine that can act as a growth factor for activated T and NK cells, enhance the lytic activity of NK/lymphokine- activated killer cells, and stimulate the production of IFN-gamma by resting PBMC; Belongs to the type I cytokine receptor family. Type 3 subfamily.
17	SLC8A1	Solute Carrier Family 8 Member A1	Solute carrier family 8 (sodium/calcium exchanger), member 1; Mediates the exchange of one Ca(2+) ion against three to four Na(+) ions across the cell membrane, and thereby contributes to the regulation of cytoplasmic Ca(2+) levels and Ca(2+)- dependent cellular processes. Contributes to Ca(2+) transport during excitation-contraction coupling in muscle. In a first phase, voltage-gated channels mediate the rapid increase of cytoplasmic Ca(2+) levels due to release of Ca(2+) stores from the endoplasmic reticulum. SLC8A1 mediates the export of Ca(2+) from the cell during the next phase, so that cytoplasmic Ca(2+) levels rapidly return to baseline. Required for normal embryonic heart development and the onset of heart contractions; Solute carriers
18	MLF1	Myeloid Leukemia Factor 1	Myelodysplasia-myeloid leukemia factor 1; Involved in lineage commitment of primary hemopoietic progenitors by restricting erythroid formation and enhancing myeloid formation. Interferes with erythropoietin-induced erythroid terminal differentiation by preventing cells from exiting the cell cycle through suppression of CDKN1B/p27Kip1 levels. Suppresses RFWD2/COP1 activity via CSN3 which activates p53 and induces cell cycle arrest. Binds DNA and affects the expression of a number of genes so may function as a transcription factor in the nucleus; Belongs to the MLF family.
19	BCL2A1	BCL2 Related Protein A1	Hemopoietic-specific early response protein; Retards apoptosis induced by IL-3 deprivation. May function in the response of hemopoietic cells to external signals and in maintaining endothelial survival during infection (By similarity). Can inhibit apoptosis induced by serum starvation in the mammary epithelial cell line HC11 (By similarity); BCL2 family
20	AMER1	APC Membrane Recruitment Protein 1	Wilms tumor gene on the X chromosome protein; Regulator of the canonical Wnt signaling pathway. Acts by specifically binding phosphatidylinositol 4,5-bisphosphate (PtdIns(4,5)P2), translocating to the cell membrane and interacting with key regulators of the canonical Wnt signaling pathway, such as components of the beta-catenin destruction complex. Acts both as a positive and negative regulator of the Wnt signaling pathway, depending on the context: acts as a positive regulator by promoting LRP6 phosphorylation. Also acts as a negative regulator by acting as a scaffold protein for the beta- catenin destruction complex and promoting stabilization of Axin at the cell membrane. Promotes CTNNB1 ubiquitination and degradation. Involved in kidney development; Belongs to the Amer family.
21	BRINP1	BMP/Retinoic Acid Inducible Neural Specific 1	Bone morphogenetic protein/retinoic acid inducible neural-specific 1; Inhibits cell proliferation by negative regulation of the G1/S transition. Mediates cell death which is not of the classical apoptotic type and regulates expression of components of the plasminogen pathway; Belongs to the BRINP family.
22	PITX2	Paired Like Homeodomain 2	RIEG bicoid-related homeobox transcription factor; Controls cell proliferation in a tissue-specific manner and is involved in morphogenesis. During embryonic development, exerts a role in the expansion of muscle progenitors. May play a role in the proper localization of asymmetric organs such as the heart and stomach. Isoform PTX2C is involved in left-right asymmetry the developing embryo (By similarity); Belongs to the paired homeobox family. Bicoid subfamily.
23	E2F5	E2F Transcription Factor 5	E2F transcription factor 5, p130-binding; Transcriptional activator that binds to E2F sites, these sites are present in the promoter of many genes whose products are involved in cell proliferation. May

			mediate growth factor- initiated signal transduction. It is likely involved in the early responses of resting cells to growth factor stimulation. Specifically required for multiciliate cell differentiation: together with MCIDAS and E2F5, binds and activate genes required for centriole biogenesis; Belongs to the E2F/DP family.
24	PDGFB	Platelet Derived Growth Factor Subunit B	Platelet-derived growth factor beta polypeptide; Growth factor that plays an essential role in the regulation of embryonic development, cell proliferation, cell migration, survival and chemotaxis. Potent mitogen for cells of mesenchymal origin. Required for normal proliferation and recruitment of pericytes and vascular smooth muscle cells in the central nervous system, skin, lung, heart and placenta. Required for normal blood vessel development, and for normal development of kidney glomeruli. Plays an important role in wound healing. Signaling is modulated by the formation of heterodimers with PDGFA (By similarity).
